# Durable hematopoiesis and tolerance after vertebral bone marrow transplant from a deceased lung transplant donor

**DOI:** 10.1172/jci.insight.198029

**Published:** 2026-02-03

**Authors:** Paul Szabolcs, Xiaohua Chen, Marian G. Michaels, Memphis Hill, Evelyn Garchar, Zarreen Amin, Heather M. Stanczak, Shawna McIntyre, Aleksandra Petrovic, Dhivyaa Rajasundaram, Ansuman Chattopadhyay, Jonathan E. Spahr, Peter D. Wearden, Geoffrey Kurland

**Affiliations:** 1 Division of Blood and Marrow Transplantation and Cellular Therapy, University of Pittsburgh Medical Center (UPMC) Children’s Hospital of Pittsburgh, University of Pittsburgh, Pittsburgh, Pennsylvania, USA.; 2 Department of Immunology, University of Pittsburgh, Pittsburgh, Pennsylvania, USA.; 3 Division of Infectious Diseases and; 4 Hematopoietic Stem Cell Laboratories, UPMC Children’s Hospital of Pittsburgh, University of Pittsburgh, Pittsburgh, Pennsylvania, USA.; 5 Department of Pediatrics, University of Washington–Seattle Children’s Hospital, Seattle, Washington, USA.; 6 Division of Health Informatics, UPMC Children’s Hospital of Pittsburgh, University of Pittsburgh, Pittsburgh, Pennsylvania, USA.; 7 Molecular Biology Information Service, Health Sciences Library System, University of Pittsburgh, Pittsburgh, Pennsylvania, USA.; 8 Division of Pulmonary Medicine, Allergy and Immunology, UPMC Children’s Hospital of Pittsburgh, University of Pittsburgh, Pittsburgh, Pennsylvania, USA.; 9 Department of Cardiovascular Services, Nemours Children’s Hospital, Orlando, Florida, USA.

**Keywords:** Clinical Research, Hematology, Immunology, Bone marrow transplantation

## Abstract

We hypothesized that bone marrow transplantation (BMT) using marrow extracted from the vertebral bodies (VBs) of an unrelated deceased lung transplant donor would be able to establish persistent hematopoiesis and generate immunity and tolerance. A teenager with severe combined immunodeficiency with lung failure due to recurrent pneumonias underwent lung transplantation in 2016 from a 1/8 HLA allele–matched unrelated donor, followed by BMT 4 months later using T cell/B cell–depleted, cryopreserved VB marrow. Rapid engraftment was followed by accelerating immune competence at 6 months, with independence from immunosuppression by 16 months. Donor T cell (>95%) and myeloid chimerism (7%–10%) has persisted for over 9 years. At 2 years after BMT, circulating T cells were hyporesponsive to host dendritic cells in vitro. T cell receptor clonotyping revealed the disappearance of host-reactive clones, and T cell RNA sequencing exhibited downmodulated signaling pathways for cytotoxicity/rejection, paired with upregulated immunomodulatory pathways, suggesting active suppression. In parallel, host monocytes upregulated certain signaling pathways, indicating active interactions between post-thymic donor T cells and host monocytes. In summary, for the first time to our knowledge, durable hematopoietic engraftment, immunity, and tolerance were demonstrable in a recipient of BMT obtained from a VB graft.

## Abstract 2

Vertebral bone marrow transplant establishes durable hemopoiesis in the first successful case, generates adaptive and innate immunity and confers tolerance between host and lung allograft.

## Introduction

Patients with primary immunodeficiency (PID) syndromes are prone to develop progressive pulmonary complications, such as bronchiectasis or interstitial lung disease (1, 2). Recurrent pneumonias caused by Gram-positive and Gram-negative bacteria and *Aspergillus* species frequently occur, regardless of their PID genotypes (3). Bone marrow transplantation (BMT) or other forms of hematopoietic stem cell transplantation (HSCT) are the most common definitive therapies to prevent progression to pulmonary failure (2). By this time, most PID patients are no longer eligible for either bilateral orthotopic lung transplant (BOLT) or HSCT due to the futility of either intervention alone. Following BOLT and solid-organ transplants (SOTs) in general, most patients require long-term immunosuppressive therapy (IST) to prevent rejection and graft loss. Despite IST, the 5-year survival rate after BOLT remains limited to 55%–60% primarily owing to chronic lung allograft dysfunction as well as complications secondary to immunosuppression (4, 5) with susceptibility to infections, malignancies, and progressive organ and metabolic dysfunctions (6). Inducing donor-specific long-term immune tolerance could eliminate the need for prolonged IST. However, it is a realistic clinical endpoint after HSCT only with sustained engraftment of donor immune cells (7), as opposed to SOT performed alone, where long-term immune tolerance is rare, occurring primarily after liver allografts (8).

Donor-specific tolerance after HSCT from the same live kidney donor has been tested over the past 2–3 decades (9–15), with full donor or persistent mixed donor chimerism from matched unrelated or half-matched (≥4 of 8) related donors, providing the most likely successful platform (16–18). When BMT was performed for hematological malignancies from sibling donors, IST-free kidney transplantation followed if the kidney was from the same sibling donor (19, 20). Strategies short of persistent donor “macrochimerism” (>4%) have failed in the unrelated or HLA-mismatched donor setting. If all leukocytes are derived from the organ donor, tolerance may be relevant only in the context of graft-versus-host (GvH) direction, while bidirectional tolerance is required in cases of mixed donor-host T cell chimerism to overcome rejection (10, 11, 13).

Successful tolerance induction to a human lung allograft was previously reported only once by Szabolcs et al. using a cryopreserved CD3^+^/CD19^+^ cell–depleted marrow graft aspirated from the iliac crest of an unrelated beating-heart donor, leading to full donor chimerism even though the patient received sub-myeloablative irradiation and chemotherapy (21).

**Figure 1:**
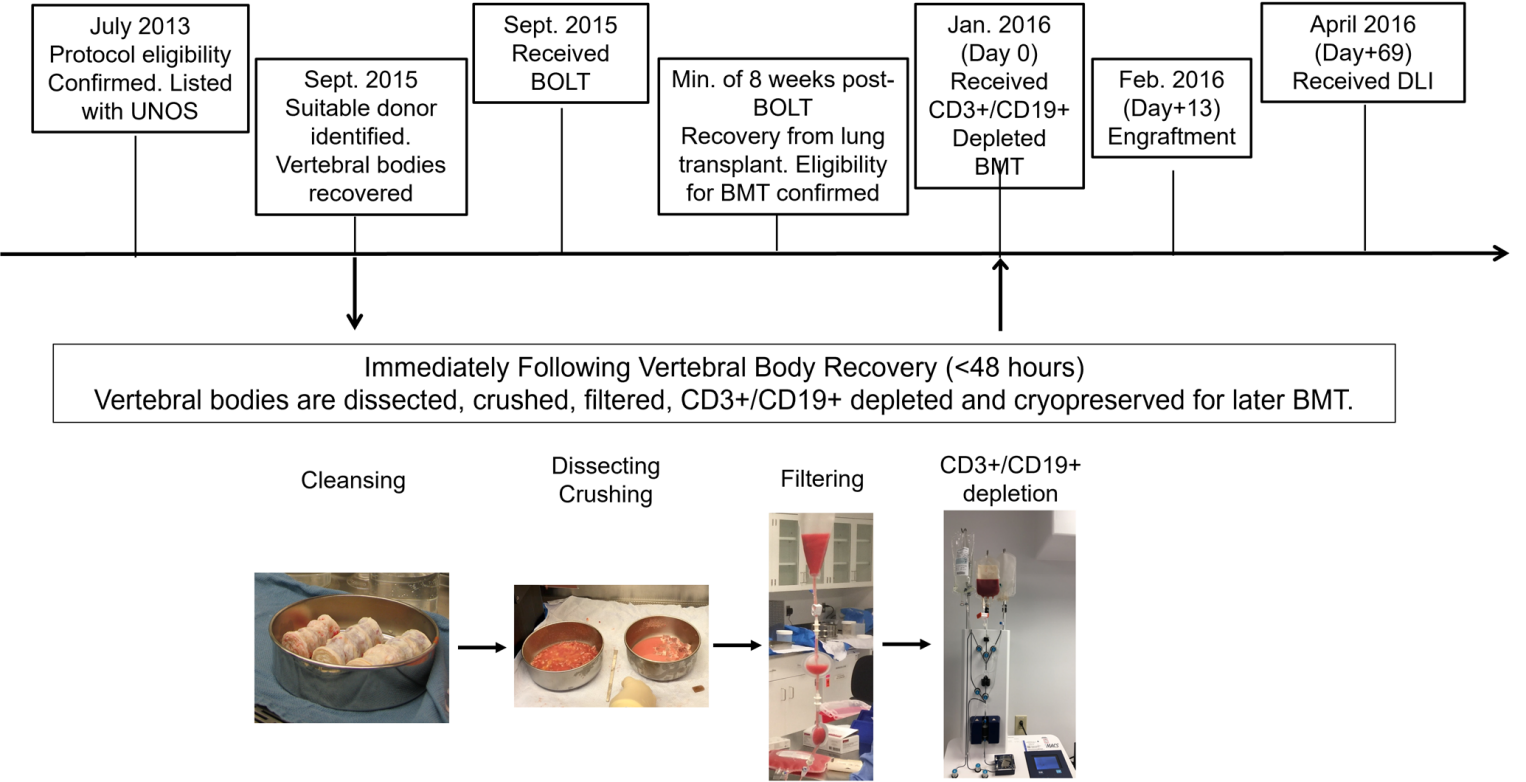
Clinical scheme and study design. After the eligibility of this study protocol was confirmed and listed with UNOS, a suitable donor was identified (July 2013 to September 2015). Vertebral bodies were surgically recovered before the patient received bilateral orthotopic lung transplant (BOLT) (September 2015). The vertebral bodies were dissected, crushed, and filtered within 48 hours after they were received. After CD3^+^ T/CD19^+^ B cell depletion using CliniMACS, vertebral bone marrow was collected and cryopreserved. A few months later (minimum of 8 weeks after BOLT), conditioning regimen was performed, followed by bone marrow transplantation (BMT) (January 2016, day 0). On day +13 after BMT (February 2016), donor engraftment was confirmed. To overcome T cell lymphopenia associated with the emergence of host T cells (73% host at 2 months), the patient received donor leukocyte infusion (DLI; April 2016, day +69). The immunosuppressive treatment was maintained until approximately 1 year after BMT.

Here we report what we believe to be the first human case of durable multilineage engraftment with vertebral bone marrow transplantation. The graft was recovered hours after cessation of circulation from the vertebral bodies of a deceased unrelated male lung allograft donor (Figure 1), and it provided protective cellular and humoral immunity for a decade despite matching at only a single HLA allele. IST-free survival without rejection or graft-versus-host disease (GvHD) established tolerance. The underlying mechanisms of long-term immune tolerance in the mixed-chimerism setting were also investigated in depth, demonstrating clonal deletion as a dominant, but not the sole, mechanism responsible for IST independence.

## Results

### Engraftment of donor graft after BMT.

**Figure 2:**
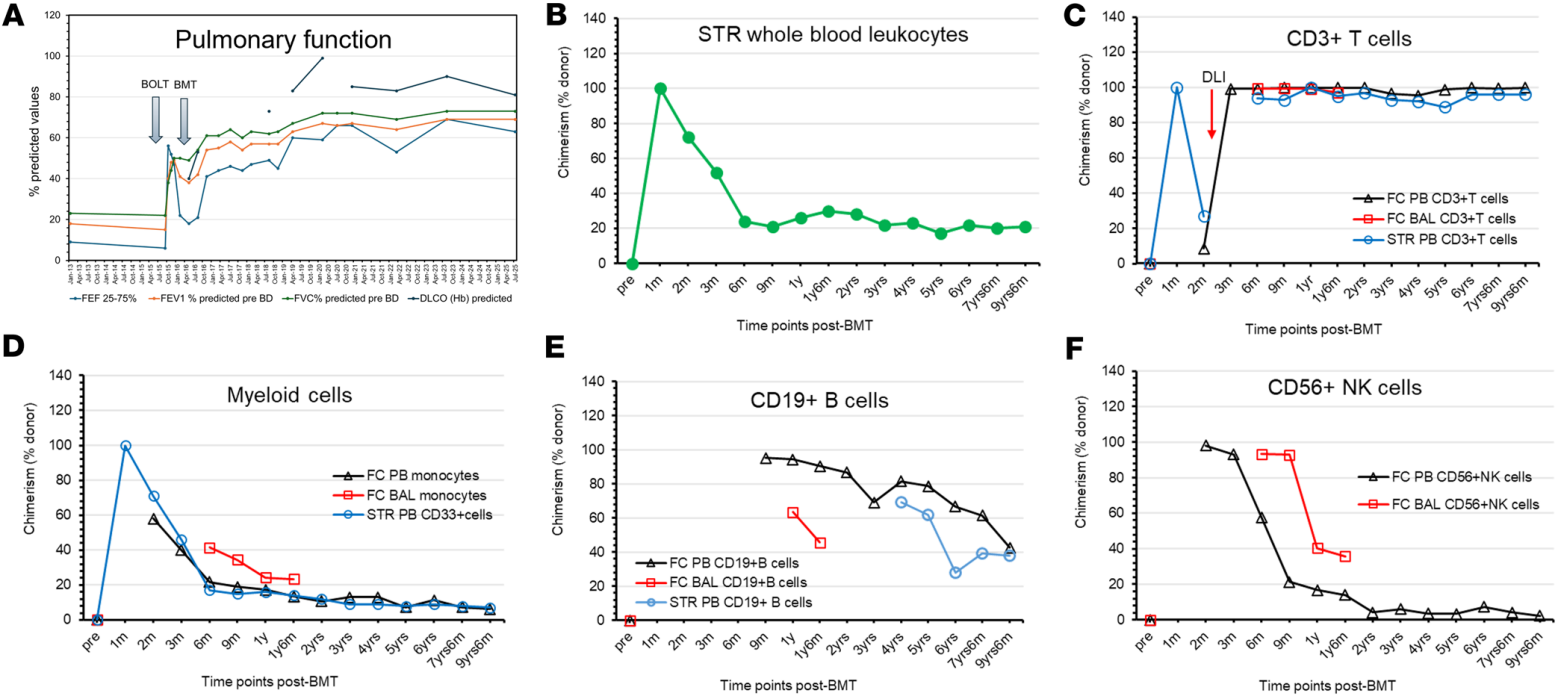
Longitudinal monitoring of donor cell chimerism and pulmonary function tests. (**A**) Pulmonary function test results prior to BOLT and over time thereafter. The *y* axis depicts percent predicted values. Arrows indicate the timing of BOLT (September 2015) and BMT (January 2016). (**B**) STR chimerism for whole peripheral blood (PB) leukocytes. (**C**) Chimerism of CD3^+^ T cells. (**D**) Chimerism of myeloid cells, including CD33^+^ cells tested by STR assay and monocytes tested by flow cytometry (FC). (**E**) Chimerism of CD19^+^ B cells. (**F**) Chimerism of CD56^+^ NK cells. The *y* axes depict percentage donor contribution at various time points after BMT, as indicated on the *x* axes. Black triangles represent donor chimerism measured by FC on PB leukocytes, red squares indicate data tested using FC on bronchoalveolar lavage (BAL), and blue circles display chimerism data tested by STR assay on purified CD3^+^ and CD33^+^ cells from PB. Green circles indicate STR assay on whole PB leukocytes. The antibodies used for FC chimerism include CD45-PerCP, CD3-BV421, CD19-BV510, and CD56-PE-Cy7 (BioLegend), along with donor-specific HLA-B12-FITC antibody and host-specific HLA-A9 biotin-conjugated antibody (both from One Lambda Solutions at Thermo Fisher Scientific).

Following neutrophil engraftment at 2 weeks, 100% donor cell chimerism was documented at 1 month from whole-blood sample, which was mostly composed of myeloid cells (Figure 2, B and D, and Supplemental Table 1; supplemental material available online with this article; https://doi.org/10.1172/jci.insight.198029DS1). To overcome T cell lymphopenia associated with the parallel emergence of host T cells (73% host at 2 months), the patient received donor leukocyte infusion (DLI) once containing about 5 × 10^4^ CD3^+^ T cells/kg from a thawed, unmodified marrow aliquot. DLI resulted in a rapid rise in donor T cell contribution (99%) (Figure 2C and Supplemental Table 1) paired with an increase in donor NK cells (93%) (Figure 2F and Supplemental Table 1) in both peripheral blood (PB) and bronchoalveolar lavage (BAL). In contrast, the monocyte fraction was only 40% donor derived (Figure 2D and Supplemental Table 1). Donor T cell chimerism above 95% (by flow cytometry) has persisted throughout 9 years (Figure 2C and Supplemental Table 1), while donor contributions to monocytes, B cells, and NK cells have gradually declined until each stabilized about 2 years after BMT and sustained beyond (7%–13%, 28%–81%, and 2%–7%, respectively) (Figure 2, D–F, and Supplemental Table 1). Serial BAL samples similarly exhibited mixed chimerism, with the last available sample, tested at 18 months after BMT, showing 97% donor-derived T cells, 24% monocytes, 46% B cells, and 36% NK cells (Figure 2, B–F, and Supplemental Table 1).

### Pulmonary status after BOLT and BMT.

Pulmonary function tests with serial spirometry demonstrated rapid improvement after BOLT, with steady parameters before and after IST withdrawal (Figure 2A) up to nearly 10 years since BOLT. Transbronchial biopsies performed 3 times after BOLT and before BMT showed no acute cellular rejection (A0), no evidence of lymphocytic bronchiolitis (B0), and no evidence of chronic/airway rejection (C0). From 5 to 25 months after BOLT, only a single biopsy (15 months after BOLT while still on tacrolimus) showed A1 (“minimal” acute cellular rejection), whereas all 3 subsequent and 7 prior lung biopsies reported no evidence of rejection (A0, B0, C0) (data not shown).

### Immune status before and after BMT.

**Figure 3:**
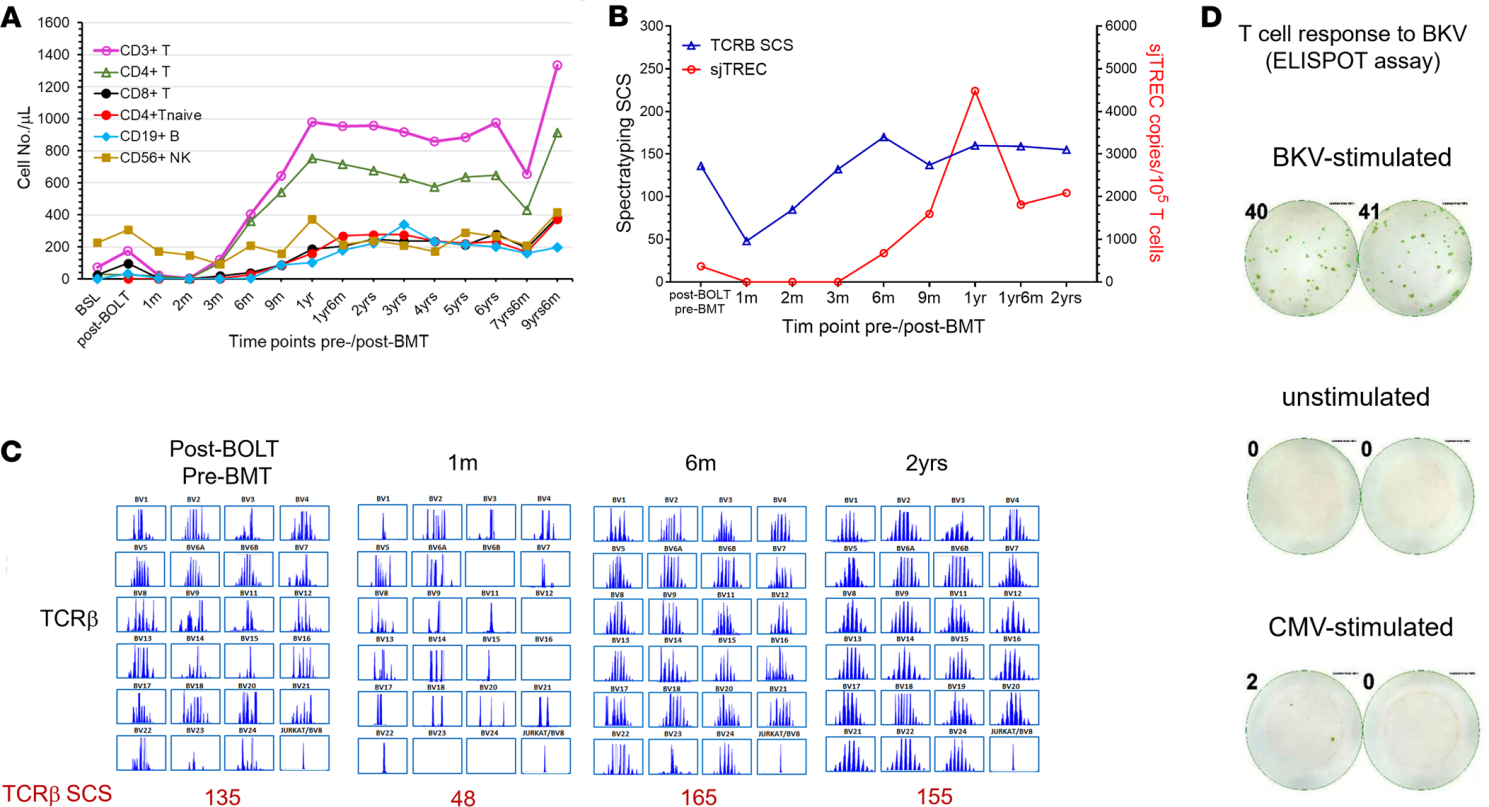
Immune reconstitution after BMT. (**A**) Numerical lymphocyte reconstitution before BMT (including baseline [BSL] and post-BOLT/pre-BMT) and during post-BMT thymopoiesis. The *y* axis shows absolute cell numbers per microliter over time (*x* axis). (**B**) Thymic output assessed by sjTREC and TCRβ spectratype over time. The left *y* axis represents the spectratype complexity score (SCS), while the right *y* axis shows sjTREC copies per 10^5^ T cells. (**C**) Acquisition of TCR repertoire diversity. Each box represents a “family” of T cell clones with a specific TCR Vβ. Individual peaks correspond to distinct TCRs based on their CDR3 length. The cumulative number of peaks is expressed as SCS, shown below each figure at the respective time point. (**D**) T cell responses illustrated by IFN-γ ELISPOT images following stimulation with overlapping peptide pools from BK virus and CMV at 6 months after BMT. Both donor and recipient were CMV negative.

The patient’s underlying IL-7 receptor–null (IL-7R–null) severe combined immunodeficiency (SCID) was characterized by progressive T cell lymphopenia before BOLT and BMT (baseline) (Figure 3A and Supplemental Table 2). T and B cell lymphopenia persisted in the early phase after BMT, reflecting the use of a CD3/CD19-depleted graft combined with lympholytic serotherapy administered peri-transplant. DLI was administered on day +68 for T cell lymphopenia (CD3^+^ T cell number < 20/μL), resulting in a rapid increase in CD3^+^ T cell numbers by day +90 after BMT (CD3^+^, 119/μL; CD4^+^, 98/μL; CD8^+^, 19/μL), with minimal impact on CD4^+^FOXP3^+^ regulatory T cell (Treg) (1.5/μL) and CD19^+^ B cell numbers (0.2/μL) (Figure 3A and Supplemental Table 2). Very mild skin GvHD involving less than 25% of body surface area appeared around 3–4 weeks after DLI but cleared within 2 weeks after administration of oral prednisone (1 mg/kg) with rapid taper plus topical therapy. Lymphopenia steadily improved over time (Figure 3A and Supplemental Table 2). Nevertheless, naive CD4^+^ T cells (CD3^+^CD4^+^CD45RA^+^CD62L^+^) remained almost undetectable (2/μL) until 6 months after BMT (25/μL), at which point thymopoiesis accelerated. Thymic output reached near-peak levels around 18 months after BMT and remained stable — and even increased — at last evaluation (Figure 3A and Supplemental Table 2).

Both signal joint T cell receptor excision circle (sjTREC) copy numbers and T cell receptor (TCR) spectratype complexity score (SCS) reached their nadir around day +30 after BMT, reflecting profound T cell depletion and use of a T cell–depleted HSCT graft. These parameters returned to pre-BMT levels around 3 months (for TCRβ SCS) and 6 months (for sjTREC), with continued acceleration peaking around 2 years after BMT (Figure 3, B and C, and Supplemental Table 2), indicating robust de novo thymopoiesis. Although CMV, EBV, and adenovirus viremia were not detected at any time, the patient exhibited BK viremia, consistent with BK virus–specific T cell activity, which was demonstrable by IFN-γ enzyme-linked immunospot (ELISPOT) assay (MilliporeSigma) at approximately 6 months after BMT (Figure 3D). Mitogen testing (pokeweed mitogen and phytohemagglutinin stimulation) revealed normal responses (data not shown). The patient established normal IgA, IgG, and IgM levels, allowing discontinuation of intravenous Ig supplementation about 16 months followed by initiation of full vaccination about 2 years after BMT. By 3 years after BMT, vaccine responses were detected against tetanus and several of the 16 tested pneumococcal serotypes, and in 2022, against SARS-CoV-2 vaccination (data not shown). Since BMT, radiographic pneumonia has not recurred, and no autoimmune events were noted.

### Hyporesponsiveness to host following systemic IST withdrawal.

**Figure 4:**
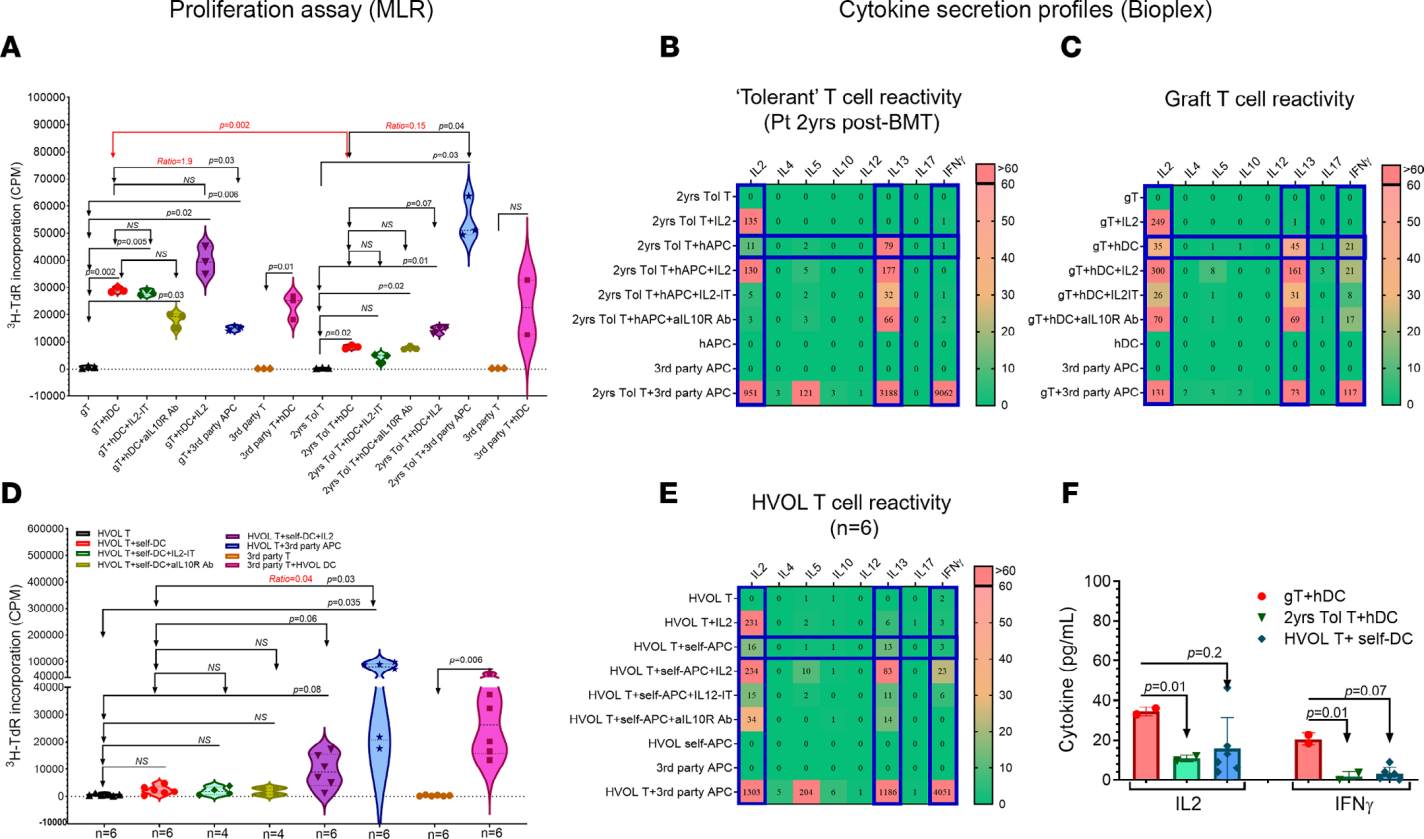
Anti-host proliferation by donor T cells in MLR and cytokine profiles contrasting circulating donor-derived T cells at tolerant state with graft T cells. (**A**) Proliferative responses of graft T (gT) cells or circulating patient T cells (2 years Tol T, all donor-derived) obtained at clinical tolerance (2 years after BMT) against host dendritic cells (hDCs), with or without in vitro modifications intended to “break tolerance,” including addition of low-dose IL-2 (purple triangles), Treg depletion using IL-2–conjugated immunotoxin (IL2-IT) (turquoise diamonds), and anti–IL-10R antibody treatment (olive hexagons). Positive controls included gT or circulating T cells tested against third-party antigen-presenting cells (APCs) and third-party T cells tested against hDCs. “Ratio” represents T+hDC/T+3rd party APC. The *y* axis shows ^3^H-thymidine incorporation counts per minute (CPM). (**B**) Cytokine profiles generated from MLR (shown in **A**) culture supernatants of circulating donor-derived T cells (2 years Tol T) at clinical tolerance (2 years after BMT), alone and stimulated with hDCs with or without modifiers. (**C**) Cytokine profiles generated from MLR (shown in **A**) culture supernatants of gT cells, alone and stimulated with hDCs with or without modifiers. (**D**) Autologous MLR assay for healthy volunteer (HVOL; *n* = 6) T cells alone and against self-DCs under the same conditions as in **A**. Significant or near-significant *P* values (≤0.05) by paired *t* test are indicated. (**E**) Cytokine profiles generated from MLR (shown in **D**) culture supernatants of HVOL T cells alone and stimulated with self-DCs with or without modifiers. The *y* axis represents in vitro culture conditions; the top *x* axis lists tested cytokines. Each square reflects the mean normalized cytokine value (pg/mL, with hDC value subtracted) for each condition. Color scale bar is shown at right. (**F**) Comparison of IL-2 and IFN-γ secretion by gT cells, circulating T cells at 2 years after BMT (2 years Tol T), and HVOL T cells, after 5-day stimulation with hDCs (for gT and 2 years Tol T) or self-DCs (for HVOL T). Paired *t* tests with Bonferroni’s multiple-comparison adjustment were used for MLR assays. Student’s *t* tests were applied to cytokine data analysis. All analyses were performed with 2-tailed tests, and results were considered statistically significant at *P* < 0.05.

At 2 years after BMT (off IST for ~8 months), circulating T cells (2 years Tol T, ~99% donor) displayed reduced proliferation against host dendritic cells (hDCs) in the mixed lymphocyte reaction (MLR) assay (Figure 3A) compared with graft T (gT) cells, isolated before BMT, which maintained robust proliferation against hDCs (*P* = 0.002) (Figure 4A). Simultaneously, circulating T cells responded vigorously to third-party antigen-presenting cells (APCs) (Figure 4A). Similarly, hDCs effectively stimulated third-party T cells (Figure 4A). When the relative ratio of anti-host proliferative capacity to anti-third-party APCs was examined, the tolerant T cells at 2 years after BMT exhibited a much lower ratio (0.15) compared with graft T cells (ratio = 1.9) (Figure 4A), demonstrating more than 10-fold diminished anti-host proliferative responses toward hDCs by the time of clinical tolerance (Tol). Cytokines detected from these MLR supernatants also reflected hyporeactivity toward the recipient (Figure 4B), mirroring T cell proliferation results (Figure 4A), and were comparable to the anti-self MLR dataset from healthy volunteers (Figure 4E). In contrast, the same T cells displayed vigorous cytokine secretion against third-party APCs, including IL-2, IL-13, and most notably IFN-γ (Figure 4B). Graft T cells, in comparison, showed robust secretion of these cytokines in response to hDCs (Figure 4C).

### Attempts to restore anti-host alloreactivity by in vitro modulations at tolerant time point.

To explore potential peripheral mechanisms associated with clinical tolerance, we depleted Tregs in vitro from the patient’s T cells before MLR assay (21). Rebound T cell proliferation was not detectable in samples drawn 2 years after BMT (Figure 4, A and B), indicating the lack of a Treg role sustaining tolerance at this stage. Similarly, the addition of anti–IL-10R blocking antibody to MLR assay, intended to interfere with Treg and type 1 regulatory T cell (Tr1 cell) functions (22), failed to enhance anti-host responses (Figure 4, A and B).

Low-dose exogenous IL-2 added to the MLR (23) to test for anergy led to a modest increase in anti-host T cell proliferation compared with cultures without it (*P* = 0.07) (Figure 4A). Nevertheless, a similar increase in proliferation was observed using the same in vitro modulation of autologous MLR from healthy volunteers (*P* = 0.06) (Figure 4D).

Neither Treg depletion nor IL-10R blockade resulted in increased IL-2 or IFN-γ levels (Figure 4B). A slight increase in IL-13 (79 pg/mL) was observed at the tolerant stage (Figure 4B). Meanwhile, low-dose IL-2 supplementation increased the secretion of IL-13 (177 pg/mL), but this level remained far below that observed in response to third-party APCs (3,160 pg/mL) (Figure 4B).

### Tracking host-reactive clones by TCRβ repertoire sequencing.

**Figure 5:**
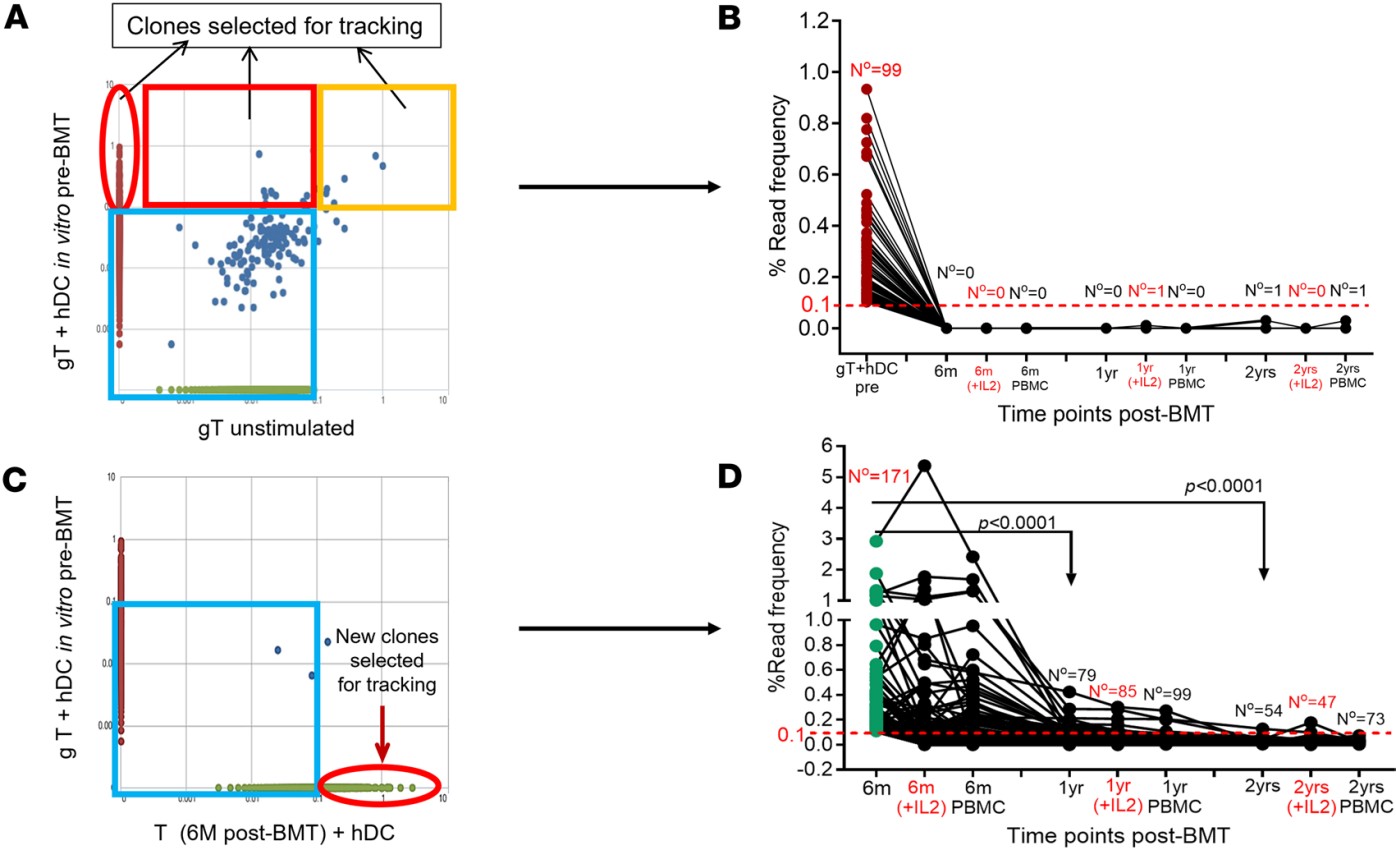
Tracking host-reactive T cell clones using Clonal Tracker tool in ImmunoSEQ Analyzer software v2. (**A**) Identification of host-reactive T cell clones based on their unique TCRβ CDR3 sequences from pre-BMT bone marrow graft (1 × 10^5^ purified T cells) after in vitro stimulation with hDCs at 5:1 ratio. Clones with a read frequency ≥ 0.1% (*y* axis) compared with unstimulated graft T cells (*x* axis) were selected for tracking. (**B**) Longitudinal tracking of host-reactive T cell clones (clonal type number = 99) in the patient after BMT. The *y* axis shows percentage read frequency of each clone type over time (*x* axis). Each dark red circle represents an individual host-reactive clone type identified among graft T cells. Addition of IL-2 to the MLR culture to reverse potential anergy is indicated in red font. (**C**) Selection of “new” alloreactive T cell clone types (green circles) that are ≥0.1% frequency and were not identifiable in gT+hDC coculture. (**D**) Tracking of these “new” alloreactive T cell clones (clonal type number = 171) after BMT. Numbers at each time point indicate the count of detectable clonal types at any frequency. The Mann-Whitney non-parametric test was used to compare clonal T cell frequencies. All analyses were performed with 2-tailed tests, and the results were considered statistically significant at *P* < 0.05.

T cell clones with anti-host reactivity (no. = 99) (Figure 5B) from the original unprocessed vertebral body (VB) marrow graft (1 × 10^5^ purified T cells) were identified after their in vitro expansion in MLR assay in response to hDCs (Figure 5A). These clones became undetectable from the circulation by 6 months after BMT (Figure 5B). All but one (at only 0.03% frequency) of these T cell clones remained undetectable after tacrolimus was fully withdrawn (Figure 5B). At 6 months after BMT, while the patient was still receiving tacrolimus, “new” host-reactive T cell clones emerged in the circulation (no. = 171) (Figure 5D). Notably, these clones were not among the original host-reactive clones present in the VB graft (Figure 5C). Most of these clones also disappeared over time, while the remaining ones became greatly diminished in read frequency and remained at very low frequency even after withdrawal of tacrolimus (Figure 5D). Supplementing the MLR microcultures with a low dose of IL-2 neither prevented the disappearance of alloreactive T cell clones (Figure 5, B and D) nor impacted the diminution of the read frequency (mean = 0.01% at 2 years) of the remaining “new” host-reactive T cell clones (no. = 54 in 2 years T+hDC, no. = 47 in 2 years T+hDC+IL-2, or no. = 73 in 2 years PBMC), arguing against a significant role for anergy (Figure 5, B and D).

### Analysis of signaling pathways in T cells and monocytes at status of clinical tolerance.

**Figure 6:**
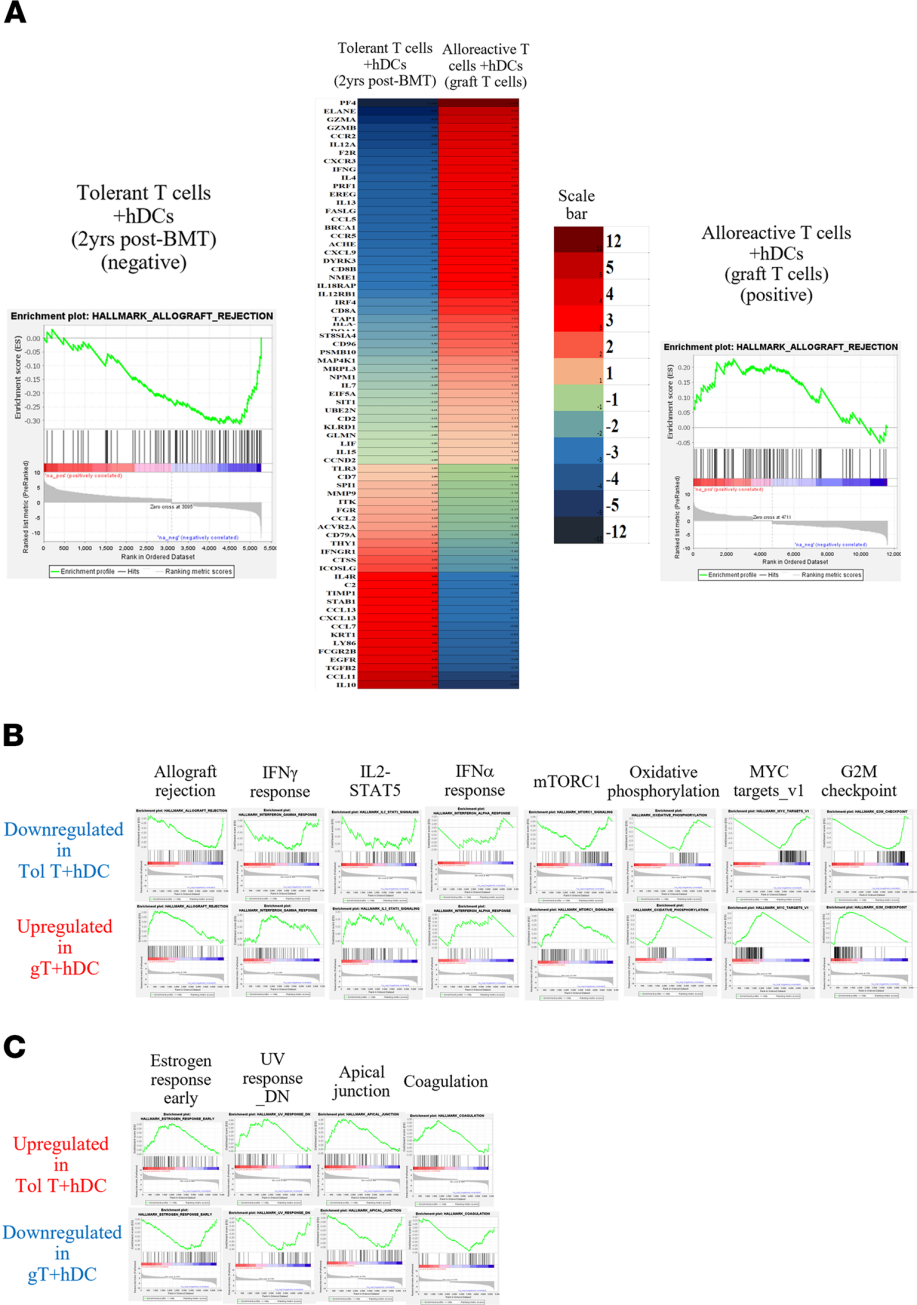
Gene Set Enrichment Analysis signaling pathway analysis comparing circulating T cells (donor origin) in tolerant state with T cells from the donor graft. (**A**) After stimulation with hDCs, the “allograft rejection” signaling pathway was downregulated in tolerant T cells (Tol T) 2 years after BMT (left enrichment plot) compared with graft T cells containing host-reactive clones before BMT (right enrichment plot). Individual genes are displayed on a heatmap, with red indicating upregulation (positive rank metric score) and blue indicating downregulation (negative rank metric score). The color scale bar is shown on the right side of the heatmap. (**B**) Eight enrichment plots showing gene expression pathways downregulated in tolerant T cells (top row) compared with graft T cells (bottom row). (**C**) Four gene expression pathways upregulated in tolerant T cells (top row) compared with graft T cells (bottom row). The genes were selected based on their false discovery rate (FDR) ≤ 0.05 and log_2_ fold changes ≥ 1 or ≤ –1. Data were analyzed using Gene Set Enrichment Analysis (GSEA) v4.3.2. Any signaling pathway was considered significantly altered if its nominal *P* value was ≤ 0.05 and/or its FDR *q* value ≤ 0.25. In **A**, relative gene expression was visualized through “conditional formatting” in Microsoft Excel.

We compared the gene expression profiles and signaling pathways of the patient’s circulating T cells (~99% donor) at the tolerant stage 2 years after BMT (Tol T) with those of T cells from the original unmodified marrow graft (gT). The comparison was conducted following in vitro stimulation with hDCs or through direct analysis of cells isolated from the circulation or from the marrow graft. Using Gene Set Enrichment Analysis (GSEA; UC San Diego, San Diego, California, USA, and Broad Institute, Cambridge, Massachusetts, USA) to compare tolerant circulating T cells at 2 years with gT cells stimulated with hDCs, we identified 22 significantly differentially expressed pathways. Among these, 15 were downregulated, exemplified by the “allograft rejection” pathway (Figure 6A and Supplemental Table 5) and the “IL2/STAT5 signaling,” “IFNγ response,” “G2M checkpoint,” “IFNα response,” and “mTORC1 signaling” pathways (Figure 6B and Supplemental Table 6). In contrast, 7 pathways were upregulated, exemplified by “estrogen response early,” “UV response DN,” “apical junction,” and “coagulation” (Figure 6C and Supplemental Table 6).

**Figure 7:**
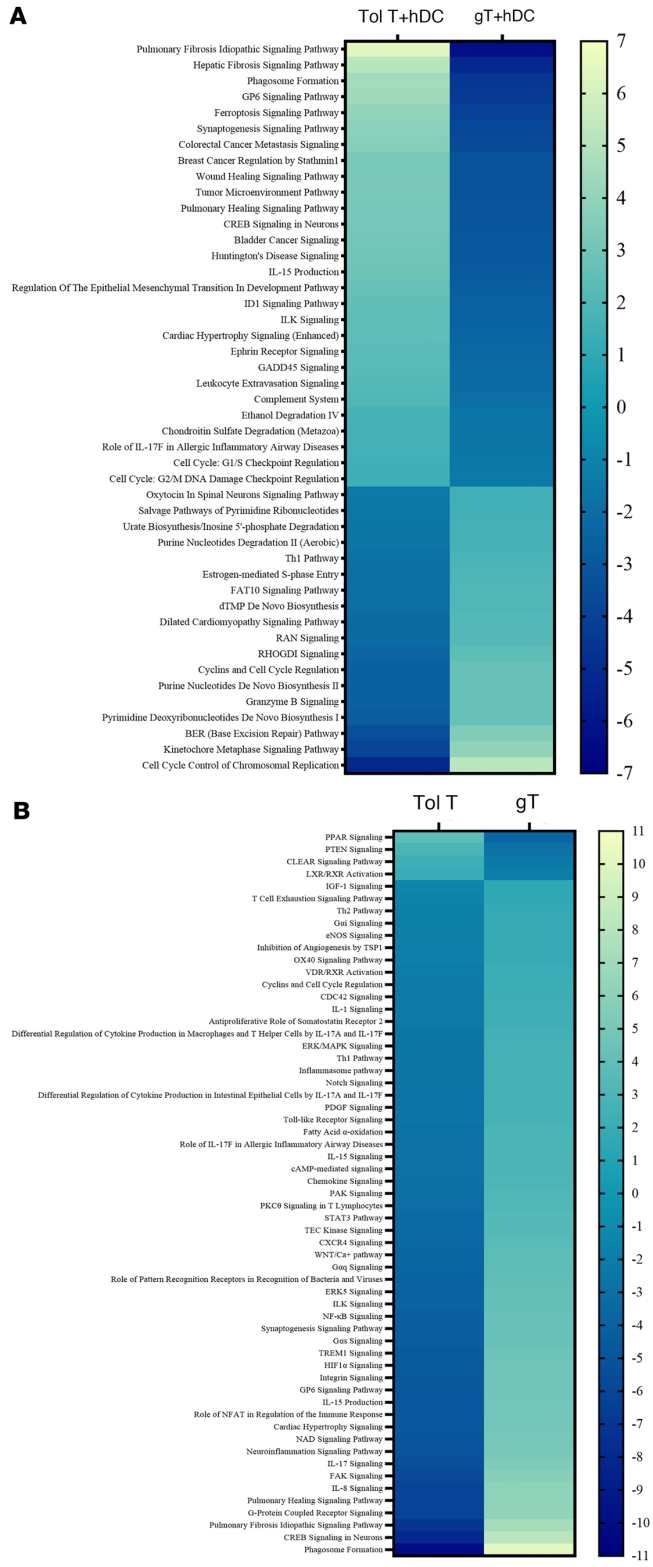
Signaling pathway analysis contrasting circulating T cells (donor origin) at tolerant state with T cells from the donor graft. (**A**) Ingenuity Pathway Analysis (IPA) comparing tolerant T cells (Tol T; 99.8% donor origin) collected 2 years after BMT with bone marrow graft T (gT) cells collected before BMT, which possessed potential alloreactivity. Both cell populations were stimulated in vitro with hDCs for 7 days. (**B**) Heatmap comparison of signaling pathways between tolerant T cells (Tol T; left column) and graft T (gT) cells (right column) without in vitro stimulation. Each box represents a signaling pathway that is either activated (positive *z* score, light yellow for most positive) or inhibited (negative *z* score, dark blue for most negative). Pathways are listed on the *y* axis, and the color scale bar is shown on the right side of the heatmap.

Using Ingenuity Pathway Analysis (IPA) software, we identified 44 pathways with significant differences, contrasting the patient’s tolerant T cells to graft T cells after each were stimulated with hDCs. Inhibited pathways (negative *z* score) were exemplified by “Th1 pathway,” “cyclins and cell cycle regulation,” and “granzyme B signaling.” Conversely, tolerant T cells exhibited activation (positive *z* score) of pathways, such as “cAMP response element–binding protein (CREB) in neurons,” “ferroptosis signaling,” and “phagosome formation” (Figure 7A and Supplemental Table 7). IPA also revealed distinct patterns of signaling pathways in the patient’s circulating tolerant T cells alone compared with graft T cells alone, despite both being of donor origin (Figure 7B and Supplemental Table 8). There was dichotomy in CREB signaling when tolerant T cells and graft T cells were compared with each other dependent on exposure to hDCs or not. Notably, CREB signaling was relatively quiescent (*z* score: –8) when Tol T were compared with gT cells without any hDC stimulation, while it became more active (*z* score: 3) in tolerant T cells compared with graft T cells after exposure to hDCs by each (Supplemental Tables 7 and 8).

**Figure 8:**
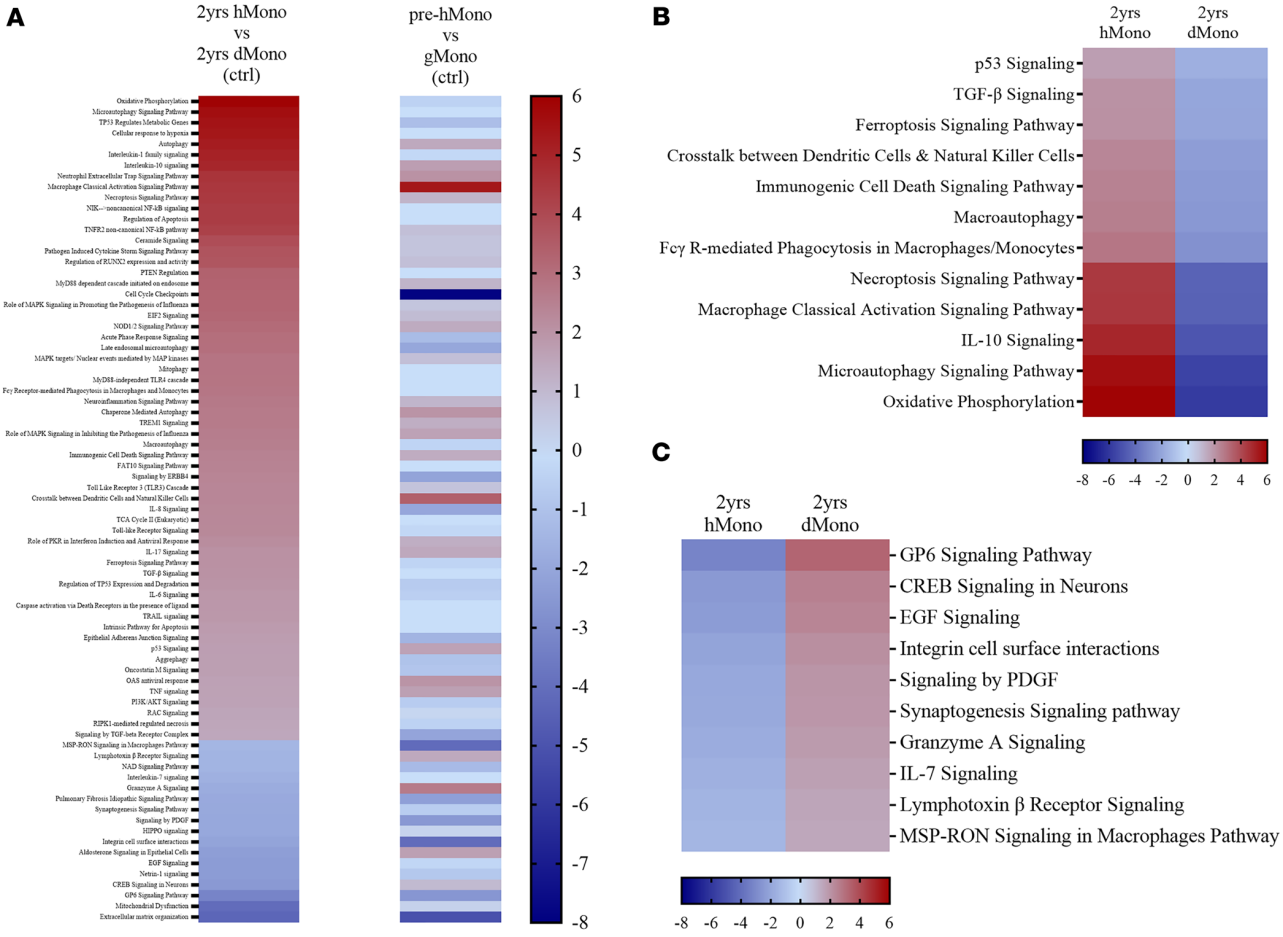
Comparison of signaling pathways between circulating host and donor monocytes from the same time point using IPA software. (**A**) Heatmap comparison of signaling pathways between host monocytes (hMono) and donor monocytes (dMono) collected either from flow cytometrically sorted monocytes using unique and HLA-specific antibodies at 2 years after BMT (left column) or from patient PB and graft before BMT (right column). Each box represents a signaling pathway either activated (positive *z* score ≥ 2, dark red as most positive) or inhibited (negative *z* score ≤ –2, dark blue as most negative). Pathways are listed on the *y* axis, and the *z* score color scale bar is shown on the right side of the heatmap. (**B**) Twelve pathways from the left column of **A** that are activated in circulating hMono (left column) relative to circulating dMono (right column) are highlighted. (**C**) Ten representative signaling pathways from the left column of **A** that are quiescent in hMono (left column) compared with dMono (right column) are highlighted.

At 2 years after BMT, the patient’s circulating CD14^+^ monocytes (~10%–11% donor by flow cytometry) were sorted for isolation of donor from host monocytes (purity: ~93% or ~100%, respectively) (Supplemental Figure 1) and were subjected to RNA sequencing (RNA-seq) analysis. Distinct patterns of the pathway activities were seen when coexisting donor and host monocytes were compared (Figure 8A, left column, and Supplemental Table 9), and these patterns differed from those observed before BMT (Figure 8A, right column). We highlighted 77 of 251 (Supplemental Table 9) pathways that showed statistically significant differences between donor and host monocytes (dMono; hMono) (Figure 8A). Circulating hMono showed relatively higher activation for several pathways, exemplified by “p53 signaling,” “TGF-β signaling,” “ferroptosis signaling,” “immunogenic cell death signaling,” “macroautophagy,” “FcγR-mediated phagocytosis in macrophages and monocytes,” “necroptosis signaling,” “macrophage classical activation signaling,” “IL-10 signaling,” and “microautophagy signaling” pathways (Figure 8B and listed in Supplemental Table 9). Conversely, dMono displayed higher activation for pathways such as “CREB signaling in neurons,” “granzyme A signaling,” and “lymphotoxin β receptor” (Figure 8C and listed in Supplemental Table 9).

## Discussion

We present what we believe to be the first successful human case of durable engraftment and immune competence in a recipient of deceased-donor vertebral bone marrow. Notably, the patient had received BOLT from the same donor a few months earlier. Persistent mixed myeloid chimerism, despite only 1 of 8 HLA match at the allele level, paired with 97%–99% T cell chimerism, has enabled rejection-free, stable pulmonary function, protective immunity, and absence of GvHD for over 8 years without IST. The VB marrow graft was recovered hours after cross-clamping and underwent extensive processing prior to cryopreservation. Durable engraftment from the VB graft was feasible despite reduced-intensity conditioning with total-body irradiation (TBI) dose approximately 6-fold lower than myeloablative antileukemic regimens, suggesting that reduced-intensity dosing will also be suitable for patients with more host T cells, while some dose reduction can still protect recipients from potential comorbidities. Protective immunity — without autoimmunity — toward past and new microbes has been sustained for over 8 years, including protective responses to vaccinations.

There have been 3 prior reports of iliac crest BMT from deceased donors. All were performed following standard aspiration technique from the iliac crest of brain-dead donors either with intact circulation or within minutes of the cessation of heart beats. Durable hematopoiesis, immune competence, and tolerance were not evaluable in the report by Blazar et al. (24) with the recipient death occurring on day +86 due to GvHD complications. In the case reported by Kapelushnik et al. (25), the donor was a related HLA-matched sibling, and the patient was followed only until 8 months after BMT, although medication-free survival was noted. The third case was our previously published report (21), which described 4 years of follow-up demonstrating sustained engraftment, immune competence, and tolerance after receipt of a T cell–depleted, cryopreserved BMT from a 4/8 HLA-matched unrelated donor. Years after undergoing BOLT and BMT, that patient was recognized to have Artemis deficiency as a radiation-sensitive type of SCID due to DCLRE1C gene mutation (26), which could explain 100% donor chimerism at all times, following a single fraction of 200 cGy TBI and a single dose of thiotepa (21). She is now more than 15 years after BMT with no change in the tolerance status, per direct communication.

The infused VB marrow graft in this report was depleted of CD3^+^ T and CD19^+^ B cells, thereby preserving hematopoietic and immune progenitors that were recognized decades ago to be biologically suitable for HSCT (27–30). A small dose of DLI, encompassing the entire donor T cell repertoire, was infused about 2 months after BMT to alleviate CD3 lymphopenia and boost donor T cell chimerism. By 6 months after BMT, new donor-derived T cells had also emigrated from the host thymus, identifiable by their broad TCR repertoire diversity, newly measurable sjTREC, and flow cytometric conformation of “recent thymic emigrants.” Reflecting the low intensity of our regimen and normal host NK cell function, recipient hematopoiesis also recovered. Nevertheless, NK cells and monocytes of donor origin have persisted at steady levels, fluctuating within the range of 7%–12%, while donor B lymphocytes have consistently remained above 60%. There was a temporary alloreactive reaction shortly after DLI, accompanied by donor T cell expansion in the periphery. This was associated with short-lived acute skin GvHD without any systemic inflammation. During this period, the patient was maintained on therapeutic levels (6–10 ng/mL) of tacrolimus. A brief course of prednisone led to rapid clearance of GvHD, while still permitting the rise in donor-derived T cell numbers and relative chimerism.

Mechanisms of tolerance were interrogated by testing of circulating T cells 2 years after BMT, when clinical tolerance had already been in effect for approximately 8 months. Host-reactive T cell clones present in the donor marrow — tracked via TCR–complementarity-determining region 3 (CDR3) usage — had all but disappeared by 6 months, except for one miniscule clone detectable at 0.03%, despite the infusion of unselected donor leukocytes about 3.5 months earlier. Even after IL-2 supplementation — to account for potential anergy — these clones remained undetectable. Disappearance of host-specific alloreactive clones led to diminished proliferation and greatly reduced Th1/Th2/IL-17 cytokine secretion against host DCs. Neither Treg depletion nor IL-10 signaling blockade succeeded in restoring anti-host reactivity. Taken together, these findings support clonal T cell deletion as the major mechanism of establishing host-specific tolerance. Deletion has previously been proposed to play a significant role in sustaining long-term immune tolerance after BMT (11, 21). Interestingly, while the patient was still on full-dose tacrolimus in this vastly HLA-mismatched setting, new host-reactive T cell clones that were not present in the original marrow graft were identified at the 6-month post-BMT time point, coinciding with the onset of thymopoiesis, contributing to the peripheral T cell pool. Most, but not all, of these “new” T cell clones also disappeared by 2 years after BMT, while the remaining ones became detectable at much lower frequencies and were unresponsive to exogenous IL-2, regarding their relative frequency.

Circulating T cells in the state of clinical tolerance exhibited quiescent gene expression profiles for “allograft rejection” gene set in bulk RNA-seq analyses in response to hDCs, and inflammatory cytokines were quiescent compared with graft T cells, which displayed robust alloreactivity. Nevertheless, certain active signaling pathways were identifiable in tolerant T cells compared with those in the original graft, suggesting that some of the de novo–generated thymocytes in the periphery may exhibit active, presumably host-restrictive suppression functions. When we queried the overwhelmingly (~90%) host-derived monocytes by RNA-seq analysis, contrasting flow-sorted hMono and dMono at the tolerant state 2 years after BMT, we found that hMono exhibited relatively higher activation of pathways associated with cell death and/or suppression of immune responses. Taken together, these findings raise the distinct possibility that circulating host monocytes may play an instructive, or at least cooperative, role in suppressing residual host-reactive T cell clones that have bypassed thymic deletion. Upregulated gene sets in host monocytes may contribute to the sustained activation of putative tolerogenic and/or suppressive donor T cell subsets.

The few RNA-seq and scRNA-seq studies — primarily performed in murine models — have focused on alloreactivity related to rejection and GvHD after transplantation, rather than on long-term tolerance. Bezie et al. (31) reported that in vitro–expanded FOXP3^+^ Tregs secrete IL-34, which influences the expressions of CSF-1R, SDC1, and PTPζ on monocyte subsets; IL-34–treated human PBMCs were shown to reduce acute GvHD in immunodeficient NSG mice. This represents an extremely artificial system, unlike our studies, which examined purified T cells without exogenous cytokine bias. Nevertheless, RNA-seq profiles from the patient described here did not show significantly increased IL-34 expression in circulating tolerant, unmanipulated, donor-derived T cells compared with graft T cells that were also free of cytokine modulation (data not shown). Similarly, there was no upregulation of CSF-1R, SDC1, and PTPζ in either host- or donor-derived monocytes at 2 years after BMT, compared with the patient’s pre-BMT levels (data not shown).

A few xenogeneic GvHD studies have performed transcriptomic analysis, but none have reported groups of mice that were free of both GvHD and immunosuppression therapy (32). One exception is the Stanford group led by Drs. Bacchetta and Roncarolo, who reported GvHD-free survival in mice; however, these mice received genetically modified “super-Treg” inocula consisting of human conventional CD4^+^ T cells transduced with lentivirus particles coding for FOXP3, resulting in FOXP3 overexpression far beyond physiological levels. These cells were infused as adoptive cell therapy, adding another layer of engineered and exogenously biased experimental model. In their unique, genetically manipulated system, transcriptomic profiling was performed; however, the authors emphasized that these FOXP3-overexpressing GvHD-suppressive cells exhibit a transcriptomic signature distinct from that of their wild-type counterparts (33).

Despite the marked difference in HLA matching between the French cohort (34) — in which patients had 8/8-matched donors — and our case, which included an only 1/8-matched donor, several immune gene expression findings remain comparable. For example, ICOS overexpression observed in our tolerant T cell analysis (data not shown) aligns with their findings in the “primary tolerant” cohort, defined as patients who had never experienced GvHD. However, in contrast to their report of CD23R upregulation, we observed CD23R downregulation in tolerant T cells. Moreover, neither proliferation nor cytokine production in response to hDCs was impacted in our tolerance assays (Figure 4) after removal of FOXP3^+^ Tregs, which appeared to be expanded in the French “primary tolerant” cohort. Nevertheless, we also observed that tolerant T cells had increased expression of ICOS, LEF1, and WNT7A compared with graft T cells — but not CD25 — which is similar to their findings in the “tolerant” cohort. However, we did not detect overexpression of TCF7, IL-10, or CD73 in our resting tolerant T cells versus resting graft T cells. Differences in these genes emerged only after both cell types were exposed to hDCs, under which conditions tolerant T cells exhibited relatively increased expression of all 3 genes — similar to the findings in the French “primary tolerant” cohort. In their study, resting PBMCs from tolerant patients were subjected to paired gene expression analyses compared with their respective donor PBMCs, which differs from our experimental conditions.

In conclusion, for the first time to our knowledge, durable engraftment and immune competence were demonstrable in a patient receiving BMT from deceased-donor vertebral bone marrow. These findings also have relevance to hematological indications, with opportunities for intensifying the conditioning regimen when the underlying condition justifies it. VB transplant with mixed chimerism was sufficient to establish an immune repertoire that permitted tolerance between recipient and lung allograft. Future studies on subsequent tolerant subjects in an ongoing trial with higher resolution of single-cell RNA-seq studies could shed further light on potentially novel active tolerogenic T cell and possibly monocyte subsets as well. Our ongoing prospective clinical trial (NCT01852370) pursues VB transplantation in other forms of PID with parallel mechanistic studies (unpublished observation).

## Methods

### Sex as a biological variable.

Since this is a single case report, sex was not considered a primary biological variable. The findings are expected to be relevant to both sexes.

### Study design.

The primary objective of this single-case protocol was to ensure durable hematopoietic engraftment from deceased-lung-donor bone marrow to establish immune competence. Clinical tolerance was defined as the absence of GvHD or lung rejection, paired with stable donor chimerism without systemic IST for at least 3 months. Initiation of IST withdrawal was permissible once the patient reached >12 months after BOLT, with no lung rejection, and no grade 2 acute or extensive chronic GvHD for more than 3 months.

Heparinized PB samples were collected at baseline (pre-BOLT), post-BOLT/pre-BMT, and at 3, 6, 9, 12, and 18 months and beyond for immune reconstitution and immune tolerance studies described below.

Engraftment was clinically quantified using short tandem repeat (STR) assay. Research flow cytometry assays used donor- and host-specific anti-HLA antibodies. Immune reconstitution was monitored by flow cytometry (35), signal joint sjTREC, and TCRβ CDR3 spectratypes (36) to assess donor-derived thymopoiesis.

In vitro tolerance in the GvH direction was tested using purified T lymphocytes to measure proliferation in MLR against hDCs, with parallel cytokine secretion profiling of MLR culture supernatants (37). TCR clonal frequencies were tracked by monitoring of individual alloreactive T cell clones in unmodified VB marrow graft and in longitudinal patient samples, based on their TCRβ V-D-J sequences. A cutoff read frequency of ≥0.1% above unstimulated T cell frequencies was set for the Clonal Tracker tool of ImmunoSEQ Analyzer software v2 (Adaptive Biotechnologies).

Mechanistic laboratory studies for tolerance required demonstration of hyporeactivity in MLR assays toward hDCs with preserved competence against third-party APCs. Removal of Tregs evaluated their role in maintaining tolerance, using IL-2–immunotoxin conjugate (IL-2 IT/denileukin diftitox) exposure prior to MLR (37). The contribution of type 1 regulatory T (Tr1) cells was assessed using an anti–IL-10R blocking antibody. Low-dose exogenous IL-2 (5 IU/mL) was used to test for possible anergy (23).

If there was active suppression by Tregs or Tr1 cells, their depletion or IL-10R blockade would revive anti-host T cell clones to proliferate and secrete IFN-γ and other cytokines. In the event of anergy, exogenous IL-2 would similarly reinvigorate host-reactive T cell clones and potentially unveil “new,” previously unrecognized clones not presented in the VB marrow. Sustained hyporeactivity that was unaffected by any of these manipulations, without upregulated gene expression, would support clonal deletion as the sole mechanism for anti-host tolerance (Supplemental Table 3).

### Human subject’s clinical course before BMT.

The patient was a 14-year-old girl at the time of BOLT (September 2015), with IL-7R–deficient SCID, characterized by 2 heterozygote mutations (c589–598 del and c993 del) in the IL-7Rα chain (Supplemental Table 4). The diagnosis was established at 9 years of age following recurrent otitis media and pneumonias associated with lymphopenia after age 5 years. T cell lymphopenia (<50–60 cells/μL), along with slightly low numbers of NK cells (~110/μL) and B cells (~110/μL), was noted upon referral to University of Pittsburgh Medical Center (UPMC) Children’s Hospital in 2013. Serum IgA (~300 mg/dL) and IgM (~1,000 mg/dL) were above the normal range while the patient was on intravenous Ig (IVIg) supplementation for inadequate responses to vaccinations. Cultures of sputa and/or bronchial lavages were positive for *Alcaligenes*, *Acinetobacter*, *Serratia*, *Pseudomonas*, and *Aspergillus fumigatus*. Progressive hypoxia led to nasal cannula oxygen supplementation, and malnutrition necessitated enteral tube feeding. At the time of listing for a lung transplant, the lung allocation score was 38. Both the United Network for Organ Sharing (UNOS) lung/marrow donor and the patient were blood type O+ and were matched serologically at 2 of 6 HLA antigens (at HLA-A 02 and HLA-DRB 11), fulfilling the minimum acceptable match by her protocol, testing HLA-A, -B, and -DRB1 loci. However, they were only 1 of 8 HLA-matched at allele-level typing (sharing HLA-A 02:01). The donor was cytomegalovirus (CMV) seronegative, while the recipient tested seropositive (presumably reflecting IVIg supplementation), as blood CMV PCR test was negative multiple times. Lung transplant was performed with standard institutional induction and maintenance therapy (basiliximab, tacrolimus, mycophenolate mofetil, and short-course glucocorticoids).

### Vertebral bone marrow transplant.

The marrow suspension prepared from the T11 to L4 vertebral bones (38) was depleted of CD3^+^ and CD19^+^ cells on a CliniMACS Plus instrument (Miltenyi Biotec) (Figure 1) and then cryopreserved along with cells from the iliac crest, which contained approximately 20-fold fewer CD34^+^ progenitor cells and total nucleated cells. At 4 months after BOLT (January 2016), the patient underwent reduced-intensity conditioning, identical to that used with our previously published case (21), consisting of 200 cGy TBI ×1, 200 mg/msq thiotepa ×1, alemtuzumab (0.5 mg/kg), and horse anti-thymocyte globulin. The CD3/CD19-depleted bone marrow (CliniMACS), containing 5 × 10^6^ CD34^+^ progenitor cells/kg and 8 × 10^4^ CD3^+^ T cells/kg, was thawed and infused on transplant day. Tacrolimus and low-dose prednisone were continued for lung rejection and GvHD prophylaxis. Irradiated granulocytes from healthy volunteer donors were infused on 4 occasions during the expected neutropenic period, until day +11 after BMT. The patient received intravenous piperacillin/tazobactam together with inhaled tobramycin daily for 2 weeks. Inpatient fungal prophylaxis consisted of a combination of voriconazole and caspofungin. Voriconazole was continued in the outpatient setting. Low-dose heparin at 100 U/kg/d and ursodiol were used for veno-occlusive disease prophylaxis. Due to persistent T cell lymphopenia (mostly host origin), the patient received UNOS donor leukocyte infusion (DLI) approximately 10 weeks after BMT (containing 5 × 10^4^ CD3^+^ T cells/kg). Tapering of tacrolimus started about 12 months after BMT, and it was fully discontinued by 16 months.

### Assessment of engraftment.

Research-grade flow cytometry was performed on surface-stained PBMCs following the manufacturer’s instructions, using CD45-PerCP (catalog 347464, BD Biosciences), CD3-BV421 (catalog 562426, BD Biosciences), CD19-BV510 (catalog 562947, BD Biosciences), and CD56-PE-Cy7 (catalog 318318, BioLegend), along with donor-specific HLA-B12-FITC antibody (catalog FH0066) and host-specific HLA-A9 biotin-conjugated antibody (catalog BIH0964) (both from One Lambda Solutions at Thermo Fisher Scientific). Streptavidin-APC (catalog 016-130-084) (Jackson ImmunoResearch Laboratories Inc.) was used as secondary antibody. Clinically reported DNA-based chimerism testing was performed by the Histocompatibility Laboratory at UPMC Lab Services using STR PCR assays on PB and immunomagnetically purified CD3^+^ and CD33^+^ cells.

### Determination of lymphocyte reconstitutions using flow cytometry.

Absolute T, B, and NK cell numbers were tested using flow cytometry TruCount assay (BD Biosciences) following the manufacturer’s instructions. The antibodies included Multitest (CD45-PerCP, CD3-FITC, CD4-APC, CD8-PE) (catalog 340499, BD Biosciences), CD19-BV421 (catalog 302234, BioLegend), CD56-PE-Cy7 (catalog 318318, BioLegend), CD16-BV510 (catalog 563830, BD Biosciences), and CD14-APC-H7 (catalog 398708, BioLegend). CD4^+^ naive T cells were examined using flow cytometry antibodies including CD3-FITC (catalog 555332, BD Biosciences), CD4-BV510 (catalog 562970, BD Biosciences), CD8-APC-H7 (catalog 560179, BD Biosciences), CD45RA-PE-Cy7 (catalog 560675, BD Biosciences), and CD62L-APC (catalog 559772, BD Biosciences), and were defined as positive for CD3/CD4/CD45RA/CD62L.

### Mixed lymphocyte reactions and Bio-Plex cytokine assay.

Modified MLR assays were performed at scheduled intervals: before BMT for graft testing, and at 3, 6, 9, 12, and 18 months as well as 2 years after BMT, using purified T cells from the patient (PtT) or the VB marrow graft (gT) and host dendritic cells (hDCs) besides IM9, a third-party commercial professional APC line (21, 37). Cryopreserved supernatants collected before ^3^H-thymidine pulse were cryopreserved and batched before testing for Th1/Th2/IL-17 cytokines, using Bio-Plex kits (Bio-Rad) following the manufacturer’s instructions.

### Examination of sjTREC and TCRβ CDR3 size spectratyping.

The sjTREC and TCRβ CDR3 size spectratyping were performed as we described previously (36). The overall complexity of TCRβ subfamilies was quantitated by the spectratype complexity score (SCS), as published (36).

### Quantification of IFN-γ–secreting T cell response using ELISPOT assay.

Enzyme-linked immunospot (ELISPOT) assay was performed according to the manufacturer’s brochure (MilliporeSigma).

### TCRβ repertoire immunosequencing and RNA sequencing.

TCRβ repertoire “immunosequencing” and bulk RNA sequencing were performed by Adaptive Biotechnologies or at the Health Sciences Sequencing Core at UPMC Children’s Hospital of Pittsburgh, respectively.

### Statistics.

Paired *t* tests with Bonferroni’s multiple-comparison adjustment were used for MLR assays (Figure 4, A and D). Student’s *t* tests were applied to the cytokine data analysis (Figure 4F). The Mann-Whitney non-parametric test was used to compare clonal T cell frequencies (Figure 5, B and D). Statistical analyses were performed using GraphPad Prism software v10 (GraphPad Software Inc.). All tests were 2-tailed, and the results were considered statistically significant when the *P* value was less than 0.05.

### Study approval.

This study was approved by the Institutional Review Board (IRB) of the University of Pittsburgh (FDA-IND 15414; clinical treatment: Pitt IRB Pro113040207; mechanistic studies: Pitt IRB PRO16010311, PRO19070076, PRO1108066), and written informed consent was obtained from all participants prior to their involvement.

### Data availability.

Clinical data are available from the corresponding author. RNA-seq raw data were deposited in the Gene Expression Omnibus database (GEO GSE305710), while MLR, Bio-Plex cytokine assay, and TCRβ-seq data are provided in XLS format. TCRβ-seq FASTA data will be made available upon request.

## Author contributions

PSz designed the study, obtained IRB and FDA approval, treated the patient, supervised vertebral bone marrow processing, designed laboratory experiments, reviewed medical and laboratory data, and wrote the manuscript. XC contributed to laboratory experimental design, supervised immune monitoring experiments, performed MLR, FACS, sjTREC, TCR spectratyping, Bio-Plex, and DNA-RNA preparations for ImmunoSEQ and RNA-seq, conducted data analysis, and wrote the first draft of the manuscript and laboratory analyses. MGM oversaw infectious disease monitoring and provided clinical antimicrobial advice. MH performed FACS, ELISPOT, sjTREC, TCR spectratyping, and MLR assays and conducted data analysis. EG contributed to sample collection and performed FACS, sjTREC, and TCR spectratyping assays. ZA contributed to sample collection and performed FACS analysis. HMS oversaw vertebral marrow processing and CD3/CD19 depletion, quality control steps, and vertebral marrow cryopreservation. SM oversaw and managed all clinical and laboratory research regulatory procedures and coordinated research distribution of samples. AP referred the patient and provided prior clinical supportive care. DR oversaw processing and contributed to interpretation of RNA-seq data. AC contributed to interpretation of IPA data and partially joined the analysis. PDW performed lung transplant surgical procedures. JES and GK coordinated pulmonary and overall medical care before and after lung transplantation. All the authors reviewed, edited, and provided final approval of the manuscript.

## Funding support

- UPMC Children’s Hospital Start Up fund (to PSz) to support laboratory research activities.

## Supplementary Material

Supplemental data

Supporting data values

## Version 1 02/03/2026

In-Press Preview

## Version 2 03/23/2026

Electronic publication
