# Supplementary material for: Durable hematopoiesis and tolerance after vertebral bone marrow transplant from a deceased lung transplant donor
Source: JCI Insight. 2026 Feb 3;11(6):e198029. doi: 10.1172/jci.insight.198029 (PMC13043083; doi:10.1172/jci.insight.198029)
Supplement: Supplemental data [file jciinsight-11-198029-s305.pdf]

Figure legend:

Supplemental Figure 1. Purity of donor and host monocytes from patient PB collected 2 years post-BMT and purified using FACS sorting. A) Gating strategy for donor and host monocytes before sorting, A1, CD14+monocyte selection; A2. singlet selection; A3. Separation of donor and host monocyte based on donor HLA-specific Ab. **B)** Purity of isolated donor monocytes. B1. CD14+monocytes; B2. Singlet selection; B3. purity of isolated donor monocytes after sorting. **C)** Purity of isolated host monocytes. C1. CD14+monocytes; C2. Singlet selection. C3. purity of host monocytes after sorting.

Supplemental Table 1. Patient chimerism post-BMT (% donor)

Supplemental Table 2. Reconstitution of immune components post-BMT prior to and during thymopoiesis (cell numbers/µL by research FC).

Supplemental Table 3. Potential outcomes and mechanistic interpretation of Tolerance in different MLR set ups

Supplemental Table 4. Clinical chronology and graft characteristics

Supplemental Table 5. List of genes and their ranks in the downregulated 'Allograft Rejection' pathway analyzed by GSEA software, contrasting tolerant T cells (Tol T) and graft T cells (gT)[1] after *in vitro* stimulation with hDC.

Supplemental Table 6. Signaling pathway profile analyzed by GSEA software comparing tolerant T cells (2 years post-BMT) and graft T cells after *in vitro* stimulation with hDCs.

Supplemental Table 7. Signaling pathway profiles analyzed by IPA software contrasting circulating donor-derived tolerant T cells (Tol T, 2 years post-BMT) and donor bone marrow graft T cells (gT) after *in vitro* stimulation with hDCs.

Supplemental Table 8. Signaling pathway profiles analyzed by IPA software contrasting tolerant T cells (Tol T, 2 years post-BMT) and bone marrow graft T cells (gT) without any *in vitro* stimulation.

Supplemental Table 9. Signaling pathway profiles analyzed by IPA software contrasting co-existing circulating host and donor monocytes at 2 years post-BMT.

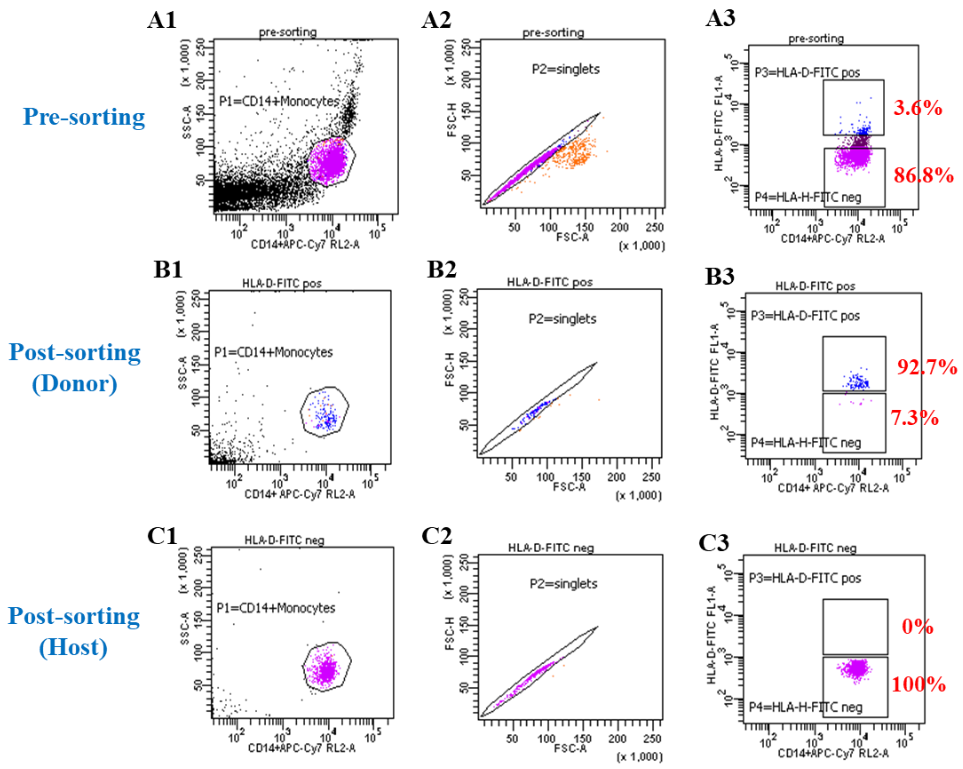

Supplemental Figure 1. Purity of donor and host monocytes from patient PB collected 2 years post-BMT and purified using FACS sorting. A) Gating strategy for donor and host monocytes before sorting, A1, CD14+monocyte selection; A2. singlet selection; A3. Separation of donor and host monocyte based on donor HLA-specific Ab. **B)** Purity of isolated donor monocytes. B1. CD14+monocytes; B2. Singlet selection; B3. purity of isolated donor monocytes after sorting. **C)** Purity of isolated host monocytes. C1. CD14+monocytes; C2. Singlet selection. C3. purity of host monocytes after sorting.

Supplemental Table 1. Patient chimerism post-BMT (% donor)

Chimerism	Post-BOLT/ pre-BMT	1m Post-BMT	2m	3m	6m	9m	1yr	1yr6m	2yrs	3yrs	4yrs	5yrs	6yrs	7yrs6m	9yrs6m
**STR[1]**															
**PB**	0	100	72	52	24	21	26	30	28	22	23	17	22	20	21
**CD3+T cells**	N/A	N/A	27	N/A	94	93	100	95	97	93	92	89	96	96	96
**CD33+ Myeloid cells**	N/A	100	71	46	17	15	16	14	12	9	9	8	9	8	7
**CD19+ B cells**											69	62	28	39	38
**FC[2] PB**															
**CD3+T cells**	N/A	N/A	8.6	99.2	99.3	99.8	99.8	99.8	99.8	96.6	95.4	98.9	99.8	99.5	99.9
**Monocyte (Myeloid)**	N/A	N/A	58	40	22	19	17.2	13.5	10.6	13.1	13	7.2	11.3	7.2	6.3
**NK cells**	N/A	N/A	98	93	58	21	17	14	4.2	6	3.6	3.3	7.3	4	2.2
**CD19+ B cells**	N/A	N/A	N/A	N/A	N/A	95	94.3	91	86.8	69	81.4	78.7	66.7	61.6	42.6
**FC[2] BAL**															
**CD3+T cells**	N/A	N/A	N/A	N/A	99.4	99.4	99.2	97.2	N/A	N/A	N/A	N/A	N/A	N/A	N/A
**Monocyte (Myeloid)**	N/A	N/A	N/A	N/A	42	34.5	24	24	N/A	N/A	N/A	N/A	N/A	N/A	N/A
**NK cells**	N/A	N/A	N/A	N/A	93	93	40	36	N/A	N/A	N/A	N/A	N/A	N/A	N/A
**CD19+ B cells**	N/A	N/A	N/A	N/A	N/A	N/A	63	46	N/A	N/A	N/A	N/A	N/A	N/A	N/A

[1]**Short Tandem Repeat (STR)** testing in the clinical UPMC HLA-laboratory performed on MiniMACS™ purified populations. (There was only 1 test that could be conducted for each assay, and no SD was displayed).
[2]**Flow Cytometry (FC)** in research laboratory. (There was only 1 test that could be conducted for each assay, and no SD was displayed).

Supplemental Table 2. Reconstitution of immune components post-BMT prior to and during thymopoiesis (cell numbers/µL by research FC)

Immune components	Baseline	Post-BLOT/pre-BMT	1m	2m	3m	6m	9m	1yr	1yr6m	2yrs	3yrs	4yrs	5yrs	6yrs	7yrs6m	9yrs6m
**CD3+T cells**	74	176	21	2.4	119	406	644	978	955	959	917	859	886	976	655	1334
**CD4+T cells**	30	25	16	2	98	361	541	753	716	676	628	574	634	646	430	912
**CD4+FOXP3+Treg**	N/A	2	N/A	N/A	1.5	5	1	6	4.4	22	4.7	8	N/A	25	N/A	N/A
**CD4+Tcon**[1]	N/A	22	N/A	N/A	90	321	487	642	619	564	596	509	N/A	445	N/A	N/A
**CD4+FOXP3+Treg/Tcon**	N/A	0.08	N/A	N/A	0.02	0.02	0.002	0.01	0.01	0.04	0.01	0.02	N/A	0.06	N/A	N/A
**CD4+T_{EM}**[2]	N/A	11	N/A	N/A	16	81	129	192	234	129	82	194	62	N/A	N/A	N/A
**CD4+T_{CM}**[3]	N/A	14	N/A	N/A	80	250	306	390	152	256	255	188	251	N/A	N/A	N/A
**CD4+Tnaive**	N/A	0.4	0	0	2	25	88	159	269	276	277	237	222	233	168	374
**CD8+T cells**	24	98	2	0.4	19	39	86	187	203	249	236	237	213	280	194	386
**CD8+T_{EM}**[4]	N/A	46	N/A	N/A	7	7	5.4	28	19	14	12.5	29	21	N/A	N/A	N/A
**CD8+T_{CM}**[5]	N/A	28	N/A	N/A	11	5	11	35	3.4	14	16	5	25	N/A	N/A	N/A
**γδT cells**	N/A	N/A	N/A	N/A	N/A	3	3	5	6	5	9	11	7	N/A	N/A	N/A
**NK T cells**	1	0.4	1.4	0	0.1	0.5	2	2	5	5	6.7	7.5	17	6.5	1.3	1.3
**NK cells**	227	308	173	149	92	208	159	373	215	240	211	173	291	268	211	417
**CD19+ B cells**	1	34	3	0.5	0.2	4	88	102	177	221	341	232	216	200	160	198
**Monocytes**	352	259	635	378	253	375	243	342	232	231	146	206	327	205	92	17.4
**pDC**[6]	6	24	35	7	6	11	7	4	13	7	11.6	8.8	10	9.7	9	7.7
**mDC**[7]	12	26	64	24	14	20	13	10	23	25	14.6	27	28.8	12	10	22
**sjTREC**[8]	N/A	369	0	0	0	677	1601	4486	1816	2085	N/A	N/A	N/A	N/A	N/A	N/A
**TCRB SCS**[9]	N/A	136	48	85	132	170	137	160	159	155	N/A	N/A	N/A	N/A	N/A	N/A

[1]**CD4+ Tcon** = CD4+ conventional T cells (CD3+CD4+CD127+CD25+/-). [2]**CD4+ T_{EM}**=CD4+ effector memory T cells (CD3+CD4+CD45RO+CD62L-). [3]**CD4+ T_{CM}**=CD4+central memory T cells (CD3+CD4+CD45RO+CD62L+). [4]**CD8+ T_{EM}**=CD8+effector memory T cells (CD3+CD8+CD45RO+CD62L-). [5]**CD8+ T_{CM}**=CD8+central memory T cells (CD3+CD8+CD45RO+CD62L+). [6]**pDC**=plasmacytoid dendritic cells. [7]**mDC**=myeloid dendritic cells. [8]**sjTREC value** = copies/1E+05 T cells; [9]**TCRB SCS** = TCRβ spectratype complexity score, maximum SCS for 23 of Vβ tested = 184. (There was only 1 test that could be conducted for each assay, and no SD was displayed).

Supplemental Table 3. Potential outcomes and mechanistic interpretation of Tolerance in different MLR set ups.

Possible mechanisms	Clonal deletion	Suppression by Treg	Anergy	Suppression by Tr1
In vitro manipulation	—	IL-2 IT*	IL-2	Anti IL-10R Ab
Proliferation in MLR	--	↑	↑	↑
Cytokine Profiling in MLR Supe	--	↑ Th1, Th2, Th17	↑ Th1, Th2, Th17	↑IL-10
TCR Immunosequencing	Absent host-reactive clones	Representation of host-reactive clones	Representation of host-reactive clones	Representation of host-reactive clones

***IL-2-IT** = IL-2-immunotoxin conjugate (Ontak™)

Supplemental Table 4. Clinical chronology and graft characteristics

Characteristics	Pt information
**Disease**	SCID (IL-7R)
**Gender**	Female
**Age at BMT (years)**	14
**HLA-match (serological)**	2/6
**HLA-match (allele level)**	1/8
**Lung Tx date**	9-25-2015
**BMT date**	1-28-2016
**BMT CD34+ Nº/kg**	5E+06
**BMT CD3+ Nº/kg**	8E+04
**DLI date**	4-5-2016
**DLI CD3 Nº/kg**	5E+04
**Stoppage date of FK506**	5-5-2017
**GvHD**	Grade I, skin
**Viral Infection**	BKV

Supplemental Table 5. List of genes and their ranks in the downregulated 'Allograft Rejection' pathway analyzed by GSEA software contrasting tolerant T cells (Tol T) and graft T cells (gT)[1] after *in vitro* stimulation with hDC.

Row No.	SYMBOL	RANK IN GENE LIST[2]	RANK METRIC SCORE[3]	RUNNING ES[4]	CORE ENRICHMENT[5]
1	IL10	82	5.941	0.0213	No
2	CCL11	200	5.144	0.0308	No
3	TGFB2	482	3.756	0.0001	No
4	EGFR	714	3.286	-0.0239	No
5	FCGR2B	832	3.084	-0.0272	No
6	LY86	912	2.918	-0.0242	No
7	KRT1	954	2.843	-0.0144	No
8	CCL7	1079	2.659	-0.0217	No
9	CXCL13	1498	2.131	-0.0889	No
10	CCL13	1513	2.117	-0.0784	No
11	STAB1	1533	2.097	-0.069	No
12	TIMP1	1541	2.085	-0.0573	No
13	C2	1635	1.99	-0.0628	No
14	IL4R	1781	1.839	-0.0793	No
15	ICOSLG	2161	1.557	-0.1426	No
16	CTSS	2210	1.523	-0.1424	No
17	IFNGR1	2316	1.447	-0.1536	No
18	THY1	2436	1.378	-0.1679	No
19	CD79A	2578	1.288	-0.1871	No
20	ACVR2A	2704	1.212	-0.2036	No
21	CCL2	2737	1.191	-0.2023	No
22	FGR	2777	1.166	-0.2026	No
23	ITK	2840	1.136	-0.2074	No
24	MMP9	2900	1.1	-0.2119	No
25	SPI1	2905	1.098	-0.2058	No
26	CD7	3021	1.039	-0.2215	No
27	TLR3	3053	1.021	-0.2211	No
28	CCND2	3185	-1.036	-0.2399	No
29	IL15	3199	-1.044	-0.2359	No
30	LIF	3218	-1.05	-0.2328	No
31	GLMN	3221	-1.051	-0.2266	No
32	KLRD1	3303	-1.082	-0.2355	No
33	CD2	3360	-1.106	-0.2394	No
34	UBE2N	3364	-1.108	-0.233	No
35	SIT1	3456	-1.139	-0.2435	No
36	EIF5A	3468	-1.146	-0.2384	No
37	IL7	3593	-1.198	-0.2549	No
38	NPM1	3647	-1.227	-0.2574	No
39	MRPL3	3722	-1.258	-0.2638	No
40	MAP4K1	3802	-1.3	-0.271	No
41	PSMB10	3926	-1.38	-0.286	No
42	CD96	3984	-1.417	-0.2882	No
43	ST8SIA4	4051	-1.467	-0.2918	No
44	HLA-DQA1	4082	-1.488	-0.2882	No
45	TAP1	4137	-1.531	-0.2891	No
46	CD8A	4254	-1.63	-0.3013	Yes
47	IRF4	4263	-1.64	-0.2926	Yes
48	IL12RB1	4356	-1.731	-0.2995	Yes
49	IL18RAP	4394	-1.785	-0.2955	Yes
50	NME1	4412	-1.814	-0.2875	Yes
51	CD8B	4461	-1.891	-0.2849	Yes
52	DYRK3	4590	-2.048	-0.2968	Yes
53	CXCL9	4647	-2.132	-0.2943	Yes
54	ACHE	4745	-2.325	-0.2985	Yes
55	CCR5	4784	-2.402	-0.2908	Yes
56	BRCA1	4792	-2.418	-0.277	Yes
57	CCL5	4857	-2.534	-0.2736	Yes
58	FASLG	4860	-2.539	-0.2581	Yes
59	IL13	4918	-2.635	-0.2526	Yes
60	EREG	4919	-2.638	-0.2362	Yes
61	PRF1	4938	-2.689	-0.2229	Yes
62	IL4	4962	-2.743	-0.2102	Yes
63	IFNG	5025	-2.949	-0.2037	Yes
64	CXCR3	5045	-3.015	-0.1886	Yes
65	F2R	5091	-3.217	-0.1771	Yes
66	IL12A	5145	-3.621	-0.1648	Yes
67	CCR2	5163	-3.895	-0.1437	Yes
68	GZMB	5166	-3.953	-0.1194	Yes
69	GZMA	5211	-4.73	-0.0984	Yes
70	ELANE	5227	-5.321	-0.0681	Yes
71	PF4	5258	-11.829	0	Yes

[1]The gT stimulated with hDC was used as a control in the comparison.
[2]**Rank in gene list**: gene rank in gene list based on their differential expression.
[3]**Rank metric score:** the value assigned to each gene in a ranked list, which determines its position within the list and is used to assess how significantly a gene is differentially expressed based on GSEA default metric (signal-to-noise ratio). The higher scores indicate genes that are more significantly differentially expressed (GSEA user guide).
[4]**Running ES**: Running enrichment score that is enrichment score at this point in the ranked list of genes.
[5]**Core enrichment:** genes with a 'Yes' value in this column contribute most to the enrichment result.

Supplemental Table 6. Signaling pathway profiles analyzed by GSEA software comparing tolerant T cells at (2 years post-BMT) and graft T cells after *in vitro* stimulation with hDCs.

2yrs Tol T+hDC vs gT+hDC (ctrl) Hallmark pathways	NES[1]	Nom p-value[2]	FDR q-value[3]	Size[4]
Downregulated (15/22 pathways)				
G2M checkpoint	-4.82	0.00E+00	0.00E+00	116
E2F targets	-4.8	0.00E+00	0.00E+00	155
MYC targets_v1	-4.23	0.00E+00	0.00E+00	102
Oxidative phosphorylation	-2.93	0.00E+00	0.00E+00	41
DNA repair	-2.81	0.00E+00	0.00E+00	38
MYC targets_v2	-2.78	0.00E+00	0.00E+00	29
Mitotic spindle	-2.77	0.00E+00	0.00E+00	68
Spermatogenesis	-2.71	0.00E+00	0.00E+00	32
Heme metabolism	-2.31	0.00E+00	1.00E-03	63
mTORC1 signaling	-2.02	0.00E+00	3.00E-03	80
Allograft rejectioon	-1.99	0.00E+00	4.00E-03	71
IFNγ response	-1.53	6.00E-03	6.10E-02	60
Unfolded protein response	-1.52	3.30E-02	6.10E-02	32
IFNα response	-1.33	9.60E-02	1.57E-01	23
IL2/STAT5 signaling	-1.22	1.33E-01	2.48E-01	66
Upregulated (7/22 pathways)				
Epithelial mesenchymal transition	2.68	0.00E+00	0.00E+00	129
Myogenesis	1.83	1.13E-03	2.38E-02	72
UV response_DN	1.81	2.32E-03	2.18E-02	49
Apical junction	1.68	1.12E-03	4.80E-02	68
Estrogen response_early	1.68	1.00E-02	3.91E-02	72
Xenobiotic metabolism	1.56	3.16E-02	9.44E-02	47
Coagulation	1.46	5.04E-02	1.71E-01	57

[1]**NES**: Normalized enrichment score which reflects the degree to which a gene set (or pathway) is concentrated at top (upregulated) or bottom (downregulated) of the ranked list of genes in the expression dataset. Higher NES value (positive or negative) suggests that the gene set is more likely to be associated with observed phenotype (GSEA user guide).
[2]**Nom *p*-value:** Nominal p-value represents statistical significance of a gene set's ES (GSEA user guide).
[3]**FDR q-value** is 'False discovery rate' that is the estimated probability that NES represents a false positive finding. GSEA recommends an FDR threshold of 0.25 or less. A lower q-value indicates a higher confidence in the significance of a gene set (GSEA user guide).
[4]**Size**: number of genes in the gene set after filtering out those genes not in the expression dataset (GSEA user guide).

Supplemental Table 7. Signaling pathway profiles analyzed by IPA software contrasting circulating donor-derived tolerant T cells (Tol T, 2 years post-BMT) and donor bone marrow graft T cells (gT) after *in vitro* stimulation.

2yrs Tol T+hDC vs gT+hDC (ctrl)[1] Ingenuity Canonical Pathways	z-score[2]	-log p-value[3]	ratio[4]
Pulmonary Fibrosis Idiopathic Signaling Pathway	6.328	5.3	0.311
Hepatic Fibrosis Signaling Pathway	5.176	2.3	0.258
Phagosome Formation	4.529	2.34	0.247
GP6 Signaling Pathway	4.352	1.4	0.274
Ferroptosis Signaling Pathway	4	2.11	0.299
Synaptogenesis Signaling Pathway	3.771	1.5	0.25
Colorectal Cancer Metastasis Signaling	3.677	1.49	0.254
Breast Cancer Regulation by Stathmin1	3.239	2.72	0.255
Wound Healing Signaling Pathway	3.222	6.11	0.34
Tumor Microenvironment Pathway	3.221	2.44	0.292
Pulmonary Healing Signaling Pathway	3.151	2.73	0.294
*CREB Signaling in Neurons	3.104	0.837	0.224
Bladder Cancer Signaling	3	3.66	0.351
Huntington's Disease Signaling	2.985	1.75	0.259
IL-15 Production	2.92	2.57	0.317
Regulation Of The Epithelial Mesenchymal Transition In Development Pathway	2.711	2	0.318
ID1 Signaling Pathway	2.517	2.41	0.286
ILK Signaling	2.496	3.85	0.318
Cardiac Hypertrophy Signaling (Enhanced)	2.449	1.53	0.24
Ephrin Receptor Signaling	2.263	2.96	0.299
GADD45 Signaling	2.236	1.93	0.339
Leukocyte Extravasation Signaling	2.16	1.37	0.259
Complement System	2	5.4	0.556
Ethanol Degradation IV	1.667	2.1	0.474
Chondroitin Sulfate Degradation (Metazoa)	1.633	1.31	0.429
Role of IL-17F in Allergic Inflammatory Airway Diseases	1.604	1.76	0.348
Cell Cycle: G1/S Checkpoint Regulation	1.528	3.33	0.388
Cell Cycle: G2/M DNA Damage Checkpoint Regulation	1.46	6.74	0.54
Oxytocin In Spinal Neurons Signaling Pathway	-1.508	1.31	0.344
Salvage Pathways of Pyrimidine Ribonucleotides	-1.512	1.55	0.292
Urate Biosynthesis/Inosine 5'-phosphate Degradation	-1.633	1.87	0.5
Purine Nucleotides Degradation II (Aerobic)	-1.633	1.57	0.421
*Th1 Pathway	-1.789	0.442	0.222
Estrogen-mediated S-phase Entry	-1.886	7.91	0.731
FAT10 Signaling Pathway	-2	5.86	0.5
dTMP De Novo Biosynthesis	-2	2.13	0.8
Dilated Cardiomyopathy Signaling Pathway	-2.117	1.65	0.277
RAN Signaling	-2.121	1.89	0.471
RHOGDI Signaling	-2.412	4.3	0.321
Cyclins and Cell Cycle Regulation	-2.646	4.32	0.398
Purine Nucleotides De Novo Biosynthesis II	-2.646	2.64	0.636
Granzyme B Signaling	-2.646	2.08	0.5
Pyrimidine Deoxyribonucleotides De Novo Biosynthesis I	-2.714	2.51	0.478
BER (Base Excision Repair) Pathway	-3.441	3.25	0.432
Kinetochore Metaphase Signaling Pathway	-3.92	13.8	0.542
Cell Cycle Control of Chromosomal Replication	-5.24	11	0.625

[1]Comparison was made of the activation status for signaling pathways between circulating T cells (donor origin) in tolerant state (Tol T) 2-years post-BMT and graft T cells (gT). Both cell types were stimulated with hDC *in vitro*, with graft T cells + hDC serving as a control. The comparison utilized RNAseq gene differential expression data sets generated by CLC Genomics Workbench software and well-established signaling pathways database from IPA software.
[2]**z-score**: IPA software calculates an 'activation z-score' for each pathway, indicating whether the pathway is likely to be activated or inhibited based on the direction of expression changes in the genes involved, as compared with the IPA database. Pathways were selected if their z-score ≥2 or ≤-2.
[3]**-log p-value**: It's a transformed version of p-value that interprets the significance of pathway. IPA uses a default threshold of -log p-value ≥1.3. The higher value holds greater significance.
[4]**Ratio**: proportion of presented dataset genes in the total number of genes in that pathway within the IPA reference set.
*These 2 pathways had z-score ≥2 or ≤-2 after hDC stimulation even though their -log p-value <1.3.

Supplemental Table 8. Signaling pathway profiles analyzed by IPA software contrasting tolerant T cells (Tol T, 2 years post-BMT) and bone marrow graft T cells (gT) without any *in vitro* stimulation.

2yrs Tol T vs gT (ctrl)[1] Ingenuity Canonical Pathways	z-score[2]	-log p-value[3]	Ratio[4]
RHOGDI Signaling	3.922	3.24	0.226
PPAR Signaling	3.71	1.48	0.212
Antioxidant Action of Vitamin C	3.638	1.86	0.224
PTEN Signaling	2.858	3.37	0.247
CLEAR Signaling Pathway	2.335	1.94	0.192
Extrinsic Prothrombin Activation Pathway	2.236	1.74	0.375
LXR/RXR Activation	1.976	4.15	0.276
IGF-1 Signaling	-1.508	1.48	0.212
Apelin Adipocyte Signaling Pathway	-1.604	1.26	0.209
T Cell Exhaustion Signaling Pathway	-1.606	1.5	0.178
Aryl Hydrocarbon Receptor Signaling	-1.789	3.55	0.252
Th2 Pathway	-1.8	7.11	0.323
Gαi Signaling	-1.8	3.02	0.243
eNOS Signaling	-1.826	2.66	0.23
Inhibition of Angiogenesis by TSP1	-1.89	3.04	0.375
OX40 Signaling Pathway	-1.89	1.6	0.189
Neuregulin Signaling	-1.941	2.3	0.235
Salvage Pathways of Pyrimidine Deoxyribonucleotides	-2	1.55	0.444
Prostanoid Biosynthesis	-2	1.38	0.4
Oleate Biosynthesis II (Animals)	-2	1.38	0.4
Pentose Phosphate Pathway	-2	1.38	0.4
Corticotropin Releasing Hormone Signaling	-2.041	1.41	0.197
VDR/RXR Activation	-2.111	2.01	0.247
Actin Nucleation by ARP-WASP Complex	-2.121	2.45	0.253
Regulation of Actin-based Motility by Rho	-2.138	2.91	0.255
Mitotic Roles of Polo-Like Kinase	-2.138	2.25	0.27
Natural Killer Cell Signaling	-2.16	2.57	0.218
Cyclins and Cell Cycle Regulation	-2.183	1.39	0.217
Epithelial Adherens Junction Signaling	-2.197	2.51	0.226
CDC42 Signaling	-2.236	3.25	0.208
Remodeling of Epithelial Adherens Junctions	-2.236	2.81	0.288
Eicosanoid Signaling	-2.236	2.11	0.262
Phenylalanine Degradation IV (Mammalian, via Side Chain)	-2.236	1.42	0.357
Gα12/13 Signaling	-2.268	1.96	0.218
Estrogen-mediated S-phase Entry	-2.333	2.14	0.346
IL-1 Signaling	-2.333	1.58	0.219
Antiproliferative Role of Somatostatin Receptor 2	-2.333	1.28	0.216
Tryptophan Degradation to 2-amino-3-carboxymuconate Semialdehyde	-2.449	5.09	1
Choline Biosynthesis III	-2.449	2.06	0.429
Differential Regulation of Cytokine Production in Macrophages and T Helper Cells by IL-17A and IL-17F	-2.449	1.49	0.333
Regulation Of The Epithelial Mesenchymal Transition In Development Pathway	-2.524	2.17	0.247
Cardiac β-adrenergic Signaling	-2.524	2.08	0.21
ERK/MAPK Signaling	-2.556	2.6	0.214
RHOA Signaling	-2.6	1.95	0.221
Th1 Pathway	-2.646	5.95	0.316
Role of IL-17A in Psoriasis	-2.646	2.81	0.5
Inflammasome pathway	-2.646	1.79	0.35
Role of NANOG in Mammalian Embryonic Stem Cell Pluripotency	-2.646	1.62	0.212
Notch Signaling	-2.646	1.53	0.27
Differential Regulation of Cytokine Production in Intestinal Epithelial Cells by IL-17A and IL-17F	-2.646	1.45	0.304
RAC Signaling	-2.668	2.3	0.226
PDGF Signaling	-2.668	1.26	0.209
Paxillin Signaling	-2.673	2.51	0.245
Toll-like Receptor Signaling	-2.673	2.01	0.247
HGF Signaling	-2.683	4.3	0.275
Nitric Oxide Signaling in the Cardiovascular System	-2.683	1.31	0.202
Macropinocytosis Signaling	-2.714	4.56	0.329
NAD biosynthesis II (from tryptophan)	-2.828	4.75	0.727
Fatty Acid α-oxidation	-2.828	3.15	0.5
Role of IL-17F in Allergic Inflammatory Airway Diseases	-2.828	1.62	0.261
Basal Cell Carcinoma Signaling	-2.84	2.12	0.257
Glioma Signaling	-2.84	1.46	0.205
Gustation Pathway	-2.874	1.28	0.185
IL-15 Signaling	-2.887	29.1	0.426
cAMP-mediated signaling	-2.887	4.23	0.238
Chondroitin Sulfate Biosynthesis (Late Stages)	-2.887	1.7	0.267
Apelin Endothelial Signaling Pathway	-2.982	1.67	0.207
Histamine Degradation	-3	5.02	0.692
Tryptophan Degradation III (Eukaryotic)	-3	3.18	0.407
Glioma Invasiveness Signaling	-3	2.8	0.282
PCP (Planar Cell Polarity) Pathway	-3	2.49	0.283
Glutathione-mediated Detoxification	-3	2.02	0.333
14-3-3-mediated Signaling	-3	1.53	0.206
Chemokine Signaling	-3	1.4	0.221
Ovarian Cancer Signaling	-3.051	2.46	0.224
Dermatan Sulfate Biosynthesis	-3.051	1.39	0.236
Pyroptosis Signaling Pathway	-3.128	2.52	0.256
Endocannabinoid Neuronal Synapse Pathway	-3.128	1.81	0.209
PAK Signaling	-3.13	4.24	0.282
P2Y Purigenic Receptor Signaling Pathway	-3.13	1.76	0.212
Crosstalk between Dendritic Cells and Natural Killer Cells	-3.138	5.36	0.33
Kinetochore Metaphase Signaling Pathway	-3.138	4.68	0.299

2yrs Tol T vs gT (ctrl)[1] Ingenuity Canonical Pathways	z-score[2]	-log p-value[3]	Ratio[4]
PKCθ Signaling in T Lymphocytes	-3.138	1.52	0.178
Osteoarthritis Pathway	-3.159	10.9	0.315
Putrescine Degradation III	-3.162	4.62	0.588
Tryptophan Degradation X (Mammalian, via Tryptamine)	-3.162	4.06	0.526
Oxidative Ethanol Degradation III	-3.162	2.31	0.345
Oxytocin In Spinal Neurons Signaling Pathway	-3.162	1.97	0.312
Phospholipases	-3.207	1.96	0.258
Sphingosine-1-phosphate Signaling	-3.266	3.49	0.265
Thrombin Signaling	-3.266	1.97	0.2
Production of Nitric Oxide and Reactive Oxygen Species in Macrophages	-3.286	1.45	0.191
Ethanol Degradation II	-3.317	3.18	0.407
FcγRIIB Signaling in B Lymphocytes	-3.317	1.67	0.229
Dermatan Sulfate Biosynthesis (Late Stages)	-3.317	1.54	0.262
Ephrin B Signaling	-3.357	2.34	0.264
Relaxin Signaling	-3.357	1.69	0.205
Ephrin Receptor Signaling	-3.411	3.52	0.234
STAT3 Pathway	-3.43	7.87	0.333
TEC Kinase Signaling	-3.43	5	0.224
Ethanol Degradation IV	-3.464	5.91	0.632
Noradrenaline and Adrenaline Degradation	-3.464	3.34	0.4
Serotonin Degradation	-3.5	2.33	0.281
B Cell Receptor Signaling	-3.528	26.9	0.368
CXCR4 Signaling	-3.53	2.79	0.229
PI3K Signaling in B Lymphocytes	-3.53	1.79	0.212
MSP-RON Signaling In Cancer Cells Pathway	-3.53	1.28	0.194
Sperm Motility	-3.545	8.57	0.287
Dopamine Degradation	-3.606	5.31	0.542
Actin Cytoskeleton Signaling	-3.651	3.77	0.229
Cell Cycle Control of Chromosomal Replication	-3.742	1.66	0.25
WNT/Ca+ pathway	-3.771	2.89	0.292
Pancreatic Adenocarcinoma Signaling	-3.771	1.61	0.21
Leukocyte Extravasation Signaling	-3.795	4.2	0.249
Gαq Signaling	-3.8	1.61	0.2
Systemic Lupus Erythematosus In T Cell Signaling Pathway	-3.812	1.65	0.178
Role of Pattern Recognition Receptors in Recognition of Bacteria and Viruses	-3.9	5.06	0.281
GNRH Signaling	-3.962	1.25	0.185
ERK5 Signaling	-4	1.86	0.243
ILK Signaling	-4.004	2.73	0.221
Regulation Of The Epithelial Mesenchymal Transition By Growth Factors Pathway	-4.012	3.14	0.229
Factors Promoting Cardiogenesis in Vertebrates	-4.017	2.31	0.223
Fcγ Receptor-mediated Phagocytosis in Macrophages and Monocytes	-4.025	1.46	0.215
Cholecystokinin/Gastrin-mediated Signaling	-4.025	1.42	0.205
HMGB1 Signaling	-4.041	3.78	0.252
Systemic Lupus Erythematosus In B Cell Signaling Pathway	-4.093	31	0.36
Xenobiotic Metabolism AHR Signaling Pathway	-4.146	2.57	0.266
Endothelin-1 Signaling	-4.226	1.71	0.199
NF-κB Signaling	-4.23	3.44	0.207
Synaptogenesis Signaling Pathway	-4.243	1.45	0.179
Gαs Signaling	-4.264	3.02	0.25
ID1 Signaling Pathway	-4.45	6.31	0.276
TREM1 Signaling	-4.49	8.61	0.431
HIF1α Signaling	-4.621	3.09	0.225
Phospholipase C Signaling	-4.629	5.61	0.22
Neurovascular Coupling Signaling Pathway	-4.629	1.81	0.197
Adrenomedullin signaling pathway	-4.644	2.53	0.216
Glioblastoma Multiforme Signaling	-4.667	4.9	0.268
Integrin Signaling	-4.667	2.31	0.21
Role of Hypercytokinemia/hyperchemokinemia in the Pathogenesis of Influenza	-4.69	2.87	0.275
GP6 Signaling Pathway	-4.707	2.7	0.242
White Adipose Tissue Browning Pathway	-4.707	1.56	0.204
IL-15 Production	-4.727	8.63	0.358
Role of NFAT in Regulation of the Immune Response	-4.727	3.97	0.212
Cardiac Hypertrophy Signaling	-4.768	1.81	0.192
Signaling by Rho Family GTPases	-4.841	5.03	0.242
NAD Signaling Pathway	-4.914	1.28	0.193
Neuroinflammation Signaling Pathway	-4.993	5.67	0.241
Wound Healing Signaling Pathway	-5.345	4.15	0.235
IL-17 Signaling	-5.376	2.69	0.223
Xenobiotic Metabolism PXR Signaling Pathway	-5.667	1.86	0.205
FAK Signaling	-5.762	7.55	0.21
IL-8 Signaling	-6.112	5.44	0.262
Pulmonary Healing Signaling Pathway	-6.147	6.85	0.284
Colorectal Cancer Metastasis Signaling	-6.181	6.7	0.261
Hepatic Fibrosis Signaling Pathway	-6.188	7.77	0.244
G-Protein Coupled Receptor Signaling	-6.237	4.93	0.2
Tumor Microenvironment Pathway	-6.272	4.91	0.264
Dendritic Cell Maturation	-6.425	7.79	0.249
Breast Cancer Regulation by Stathmin1	-6.755	5.54	0.211
Pulmonary Fibrosis Idiopathic Signaling Pathway	-7.18	6.34	0.245
Cardiac Hypertrophy Signaling (Enhanced)	-7.298	6.61	0.223
CREB Signaling in Neurons	-8.171	6.01	0.213
Phagosome Formation	-10.171	15.1	0.257

[1]Comparison was made of the activation status for signaling pathways between circulating T cells (donor origin) in tolerant state (Tol T) 2-years post-BMT and graft T cells (gT), without hDC *in vitro* stimulation. Graft T cells served as a control. The comparison utilized RNAseq gene differential expression data sets generated by CLC Genomics Workbench software and well-established signaling pathways database from IPA software.
[2]**z-score**: IPA software calculates an 'activation z-score' for each pathway, indicating whether the pathway is likely to be activated or inhibited based on the direction of expression changes in the genes involved, as compared with the IPA. Pathways were selected if their z-score ≥2 or ≤-2.
[3]**-log p-value**: It's a transformed version of p-value that interprets the significance of pathway. IPA uses a default threshold of -log p-value ≥1.3. The higher value holds greater significance.
[4]**Ratio**: proportion of presented dataset genes in the total number of genes in that pathway within the IPA reference set.

Supplemental Table 9. Signaling pathway profiles analyzed by IPA software contrasting co-existing circulating host and donor monocytes at 2 years post-BMT.

2yrs hMono vs 2yrs dMono (ctrl)[1] Ingenuity Canonical Pathways	z-score[2]	-log p-value[3]	Ratio[4]
Neutrophil degranulation	11.415	39.7	0.54
SRP-dependent cotranslational protein targeting to membrane	8.102	29.9	0.772
Eukaryotic Translation Initiation	7.398	25.3	0.713
Eukaryotic Translation Elongation	7.171	21.9	0.737
Selenoamino acid metabolism	7.14	19.6	0.682
Eukaryotic Translation Termination	7.034	20.5	0.723
Response of EIF2AK4 (GCN2) to amino acid deficiency	6.905	21.1	0.709
Nonsense-Mediated Decay (NMD)	6.333	22.3	0.692
Oxidative Phosphorylation	5.899	18	0.664
Microautophagy Signaling Pathway	5.612	7.48	0.459
TP53 Regulates Metabolic Genes	5.461	9.88	0.58
Cellular response to hypoxia	5.376	6.54	0.533
Electron transport, ATP synthesis, and heat production by uncoupling proteins	5.237	20.7	0.656
Autophagy	5.222	5.38	0.397
Class I MHC mediated antigen processing and presentation	5.157	10.4	0.411
Interleukin-1 family signaling	5.077	6.21	0.457
Cachexia Signaling Pathway	5.058	4.74	0.356
Major pathway of rRNA processing in the nucleolus and cytosol	5.051	14.6	0.527
Neddylation	5.027	5.4	0.388
Interleukin-10 signaling	4.95	9.67	0.711
Degradation of beta-catenin by the destruction complex	4.824	8.05	0.538
Mitotic Metaphase and Anaphase	4.796	5.45	0.391
Antigen Presentation Pathway	4.796	5.24	0.605
Regulation of mitotic cell cycle	4.743	4.35	0.455
Mitochondrial protein import	4.596	4.6	0.5
Neutrophil Extracellular Trap Signaling Pathway	4.585	11.5	0.418
Macrophage Classical Activation Signaling Pathway	4.5	3.16	0.367
Iron uptake and transport	4.491	2.2	0.414
Hedgehog ligand biogenesis	4.459	5.41	0.523
Necroptosis Signaling Pathway	4.45	2.52	0.359
KEAP1-NFE2L2 pathway	4.427	3.95	0.44
Regulation of Apoptosis	4.352	7.69	0.623
NIK-->noncanonical NF-kB signaling	4.352	5.91	0.55
CGAS-STING Signaling Pathway	4.341	2.99	0.381
Sphingolipid metabolism	4.243	1.85	0.368
Synthesis of DNA	4.23	3.2	0.395
TNFR2 non-canonical NF-kB pathway	4.218	6.95	0.559
Protein Sorting Signaling Pathway	4.007	5.08	0.407
COPII-mediated vesicle transport	4.004	3.78	0.458
C-type lectin receptors (CLRs)	3.906	11.8	0.531
RAB geranylgeranylation	3.9	3.16	0.446
Ceramide Signaling	3.893	5.15	0.473
Metabolism of polyamines	3.889	5.57	0.542
DNA Replication Pre-Initiation	3.833	4.53	0.442
Hedgehog 'on' state	3.812	5.97	0.5
Response to elevated platelet cytosolic Ca2+	3.81	4.05	0.409
Hepatic Cholestasis	3.787	1.44	0.313
ISG15 antiviral mechanism	3.78	2.13	0.394
Detoxification of Reactive Oxygen Species	3.771	2.66	0.486
Pathogen Induced Cytokine Storm Signaling Pathway	3.714	3.54	0.341
MyD88:MAL(TIRAP) cascade initiated on plasma membrane	3.71	3.22	0.5
Peroxisomal protein import	3.674	1.7	0.381
Regulation of RUNX2 expression and activity	3.667	4.93	0.493
Signaling by NOTCH4	3.647	8.93	0.573
NAFLD Signaling Pathway	3.622	4.55	0.382
Transcriptional regulation by RUNX3	3.507	6.12	0.49
Cargo concentration in the ER	3.5	2.24	0.471
MHC class II antigen presentation	3.464	2.86	0.381
Mitochondrial translation	3.452	2.43	0.385
E3 ubiquitin ligases ubiquitinate target proteins	3.413	5.9	0.564
RHO GTPases Activate WASPs and WAVEs	3.411	5.13	0.611
Protein ubiquitination	3.357	2.45	0.5
Fc epsilon receptor (FCERI) signaling	3.347	3.89	0.374
ABC-family proteins mediated transport	3.328	7.62	0.515
PTEN Regulation	3.328	7.27	0.46
MyD88 dependent cascade initiated on endosome	3.3	3.89	0.581
Regulation of mRNA stability by proteins that bind AU-rich elements	3.3	1.72	0.409
Cell Cycle Checkpoints	3.266	3.61	0.353
Role of MAPK Signaling in Promoting the Pathogenesis of Influenza	3.244	2.33	0.373
EIF2 Signaling	3.204	25.2	0.595
S Phase	3.202	3.93	0.43
NRF2-mediated Oxidative Stress Response	3.162	4	0.374
Cristae formation	3.153	3.3	0.548
Endosomal Sorting Complex Required For Transport (ESCRT)	3.153	3.3	0.548
NOD1/2 Signaling Pathway	3.098	2.32	0.347
Cytoprotection by HMOX1	3.053	4.48	0.5
Actin Nucleation by ARP-WASP Complex	3.053	4.33	0.451
MyD88 cascade initiated on plasma membrane	3.051	2.37	0.52
Mitotic G1 phase and G1/S transition	3	6.74	0.466
Differential Regulation of Cytokine Production in Macrophages and T Helper Cells by IL-17A and IL-17F	3	1.64	0.5
Protein Ubiquitination Pathway	2.997	9.3	0.429
Acute Phase Response Signaling	2.994	4.41	0.391
Late endosomal microautophagy	2.982	3.77	0.559
Signaling by ROBO receptors	2.941	4.86	0.44

2yrs hMono vs 2yrs dMono (ctrl)[1] Ingenuity Canonical Pathways	z-score[2]	-log p-value[3]	Ratio[4]
The citric acid (TCA) cycle and respiratory electron transport	2.921	5.33	0.545
Agrin Interactions at Neuromuscular Junction	2.887	3.54	0.456
Apelin Adipocyte Signaling Pathway	2.887	1.44	0.349
HMGB1 Signaling	2.885	4.73	0.409
TCR signaling	2.869	12.1	0.556
RAS processing	2.84	3.82	0.625
Mitophagy	2.84	2.45	0.5
Signaling by CSF3 (G-CSF)	2.84	2.45	0.5
MAPK targets/ Nuclear events mediated by MAP kinases	2.84	1.97	0.455
MyD88-independent TLR4 cascade	2.837	4.24	0.568
Signaling by Insulin receptor	2.828	1.83	0.419
Fcγ Receptor-mediated Phagocytosis in Macrophages and Monocytes	2.795	2.71	0.398
Transcriptional regulation by RUNX1	2.794	4.51	0.408
Cargo recognition for clathrin-mediated endocytosis	2.777	3.06	0.4
Signaling by the B Cell Receptor (BCR)	2.734	2.94	0.365
Neuroinflammation Signaling Pathway	2.714	4.8	0.365
Glutathione Redox Reactions I	2.714	2.05	0.5
CDP-diacylglycerol Biosynthesis I	2.714	1.58	0.444
Chaperone Mediated Autophagy	2.673	3.71	0.636
Remodeling of Epithelial Adherens Junctions	2.673	2.32	0.409
TREM1 Signaling	2.667	5.1	0.5
Alpha-protein kinase 1 signaling pathway	2.646	2.06	0.636
Pexophagy	2.646	2.06	0.636
Amyloid fiber formation	2.646	1.94	0.384
RAF/MAP kinase cascade	2.626	5.3	0.382
Role of MAPK Signaling in Inhibiting the Pathogenesis of Influenza	2.6	1.44	0.355
Protein folding	2.592	4.18	0.439
Deubiquitination	2.574	5.75	0.388
Folate Signaling Pathway	2.558	1.76	0.393
Macroautophagy	2.556	3.56	0.462
Regulation of Actin-based Motility by Rho	2.535	3.17	0.4
Glycerophospholipid biosynthesis	2.535	1.69	0.354
Superpathway of Cholesterol Biosynthesis	2.5	4.03	0.607
Signaling by FGFR3	2.5	1.48	0.4
Cholesterol biosynthesis	2.496	2.37	0.52
Multiple Sclerosis Signaling Pathway	2.492	2.3	0.338
Mitotic Prophase	2.48	5.05	0.456
Immunogenic Cell Death Signaling Pathway	2.475	2.1	0.381
COPI-mediated anterograde transport	2.469	3.47	0.416
Nucleotide Excision Repair	2.466	2.43	0.385
FAT10 Signaling Pathway	2.449	5.9	0.564
Ephrin Receptor Signaling	2.449	3.06	0.358
Endocannabinoid Cancer Inhibition Pathway	2.449	3.05	0.377
NLR signaling pathways	2.449	2.43	0.429
Pentose Phosphate Pathway	2.449	1.65	0.6
Signaling by ERBB4	2.4	1.87	0.397
Toll Like Receptor 3 (TLR3) Cascade	2.357	3.65	0.562
Crosstalk between Dendritic Cells and Natural Killer Cells	2.353	1.79	0.363
Signaling by NTRK1 (TRKA)	2.335	2.08	0.383
Advanced glycosylation endproduct receptor signaling	2.333	2.92	0.692
Vitamin-C Transport	2.333	1.32	0.435
IL-8 Signaling	2.321	3.89	0.374
Insertion of tail-anchored proteins into the endoplasmic reticulum membrane	2.309	2.44	0.545
TCA Cycle II (Eukaryotic)	2.309	2.44	0.545
Phosphatidylglycerol Biosynthesis II (Non-plastidic)	2.309	1.71	0.448
Energy dependent regulation of mTOR by LKB1-AMPK	2.309	1.33	0.414
TAK1-dependent IKK and NF-kappa-B activation	2.294	3.85	0.523
Toll-like Receptor Signaling	2.294	3.49	0.442
Senescence-Associated Secretory Phenotype (SASP)	2.268	1.94	0.384
MAP kinase activation	2.236	3.27	0.513
Role of PKR in Interferon Induction and Antiviral Response	2.188	3.45	0.394
Cholesterol Biosynthesis I	2.121	2.17	0.615
Cholesterol Biosynthesis II (via 24,25-dihydrolanosterol)	2.121	2.17	0.615
Cholesterol Biosynthesis III (via Desmosterol)	2.121	2.17	0.615
Gene Silencing by RNA	2.121	1.3	0.337
Amino acids regulate mTORC1	2.117	3.81	0.491
Interconversion of nucleotide di- and triphosphates	2.111	1.91	0.5
IL-17 Signaling	2.066	1.95	0.335
Ferroptosis Signaling Pathway	2.064	2.52	0.37
Mitotic G2-G2/M phases	2.063	10.4	0.472
Regulation of lipid metabolism by PPARalpha	2.058	1.31	0.328
TGF-β Signaling	2.043	1.39	0.34
Clathrin-mediated endocytosis	2.023	4.72	0.426
MTOR signalling	2	3.4	0.571
Glutathione Redox Reactions II	2	2.36	1
Regulation of TP53 Expression and Degradation	2	1.59	0.41
Zymosterol Biosynthesis	2	1.39	0.667
fMLP Signaling in Neutrophils	1.98	7.47	0.481
XBP1(S) activates chaperone genes	1.964	2.31	0.438
Induction of Apoptosis by HIV1	1.961	2.66	0.429
Cellular response to heat stress	1.961	2.08	0.371
Th1 Pathway	1.953	8.6	0.513
Gluconeogenesis I	1.941	2.37	0.52
Thrombin signalling through proteinase activated receptors (PARs)	1.941	1.34	0.406

2yrs hMono vs 2yrs dMono (ctrl)[1] Ingenuity Canonical Pathways	z-score[2]	-log p-value[3]	Ratio[4]
RAB GEFs exchange GTP for GDP on RABs	1.915	1.95	0.371
IL-6 Signaling	1.905	5.44	0.442
Caspase activation via Death Receptors in the presence of ligand	1.897	2.69	0.625
Valine Degradation I	1.897	2.31	0.55
TRAIL signaling	1.89	3.34	0.875
CSDE1 Signaling Pathway	1.877	2.94	0.455
TCF dependent signaling in response to WNT	1.812	4.75	0.392
Transcriptional Regulation by VENTX	1.807	1.83	0.425
Intrinsic Pathway for Apoptosis	1.8	2.94	0.455
Angiopoietin Signaling	1.789	2.25	0.395
RHOA Signaling	1.769	3.51	0.402
EPH-Ephrin signaling	1.761	3.47	0.424
Cholecystokinin/Gastrin-mediated Signaling	1.761	2.54	0.376
Epithelial Adherens Junction Signaling	1.756	8.31	0.471
Role of p14/p19ARF in Tumor Suppression	1.732	1.33	0.414
p53 Signaling	1.706	1.76	0.357
Oncostatin M Signaling	1.698	2.2	0.442
Aggrephagy	1.698	1.5	0.395
Circadian Clock	1.698	1.31	0.362
Apelin Endothelial Signaling Pathway	1.677	3.1	0.381
OAS antiviral response	1.633	1.94	0.667
Pathogenesis of Multiple Sclerosis	1.633	1.94	0.667
TNF signaling	1.616	5.45	0.544
TBC/RABGAPs	1.606	2.07	0.432
UVC-Induced MAPK Signaling	1.606	1.96	0.412
PI3K/AKT Signaling	1.605	4.84	0.394
Transcriptional Regulation by NPAS4	1.604	1.35	0.4
Translocation of SLC2A4 (GLUT4) to the plasma membrane	1.569	1.57	0.366
RAC Signaling	1.54	5.91	0.445
Sertoli Cell-Germ Cell Junction Signaling Pathway (Enhanced)	1.539	6.54	0.408
MicroRNA Biogenesis Signaling Pathway	1.522	4.79	0.399
IL-17A Signaling in Gastric Cells	1.508	2.18	0.5
Dopamine Degradation	1.508	1.59	0.458
Glycolysis I	1.508	1.59	0.458
RIPK1-mediated regulated necrosis	1.5	2.58	0.5
Wound Healing Signaling Pathway	1.463	2.26	0.332
Signaling by TGF-beta Receptor Complex	1.46	2.43	0.434
MSP-RON Signaling in Macrophages Pathway	-1.474	3.19	0.397
Glioblastoma Multiforme Signaling	-1.511	3.34	0.375
Breast Cancer Regulation by Stathmin1	-1.516	2.59	0.308
Lymphotoxin β Receptor Signaling	-1.528	2.68	0.444
NAD Signaling Pathway	-1.543	1.57	0.331
HER-2 Signaling in Breast Cancer	-1.565	6	0.404
Ribonucleotide Reductase Signaling Pathway	-1.576	2.49	0.353
ATM Signaling	-1.667	4.81	0.455
Interleukin-7 signaling	-1.667	1.61	0.476
BBSome Signaling Pathway	-1.69	2	0.304
Erythropoietin Signaling Pathway	-1.692	2.4	0.351
RUNX1 regulates megakaryocyte differentiation and platelet function	-1.698	2.55	0.431
G alpha (12/13) signalling events	-1.768	2.47	0.4
Huntington's Disease Signaling	-1.769	7.15	0.401
Orexin Signaling Pathway	-1.789	2.78	0.345
Granzyme A Signaling	-1.808	5.78	0.521
Parkinson's Signaling Pathway	-1.857	6.9	0.395
Formation of WDR5-containing histone-modifying complexes	-1.886	1.95	0.429
Signaling by SCF-KIT	-1.886	1.83	0.419
Pulmonary Fibrosis Idiopathic Signaling Pathway	-1.91	2.07	0.317
Synaptogenesis Signaling Pathway	-1.925	5.13	0.369
ROBO SLIT Signaling Pathway	-1.941	4.26	0.419
HIPPO signaling	-1.964	2.49	0.395
Signaling by PDGF	-1.964	1.31	0.362
Integrin cell surface interactions	-2.121	2.01	0.376
Spliceosomal Cycle	-2.132	2.43	0.434
Small Cell Lung Cancer Signaling	-2.132	1.58	0.351
Aldosterone Signaling in Epithelial Cells	-2.335	3.09	0.369
Netrin-1 signaling	-2.4	2.07	0.42
Sensory processing of sound by outer hair cells of the cochlea	-2.4	1.56	0.382
EGF Signaling	-2.4	1.47	0.375
CREB Signaling in Neurons	-2.514	2.12	0.301
Adrenergic Receptor Signaling Pathway (Enhanced)	-2.54	1.59	0.321
Sensory processing of sound by inner hair cells of the cochlea	-2.6	1.47	0.362
Cilium Assembly	-2.65	8.51	0.446
NCAM signaling for neurite out-growth	-2.711	1.6	0.377
Histone Modification Signaling Pathway	-2.714	3.07	0.34
Neuropathic Pain Signaling in Dorsal Horn Neurons	-2.744	1.41	0.34
GP6 Signaling Pathway	-3.244	1.46	0.333
RHO GTPase cycle	-3.298	10.3	0.396
Coronavirus Pathogenesis Pathway	-3.313	7.56	0.438
Docosahexaenoic Acid (DHA) Signaling	-3.534	2.48	0.336
Neurexins and neuroligins	-3.71	1.39	0.368
Acetylcholine Receptor Signaling Pathway	-3.81	1.73	0.326
Mitochondrial Dysfunction	-4.11	14.7	0.456
Extracellular matrix organization	-4.333	1.46	0.34

[1]A comparison of the activation status of signaling pathways between host-monocytes (hMono) and donor monocytes (dMono) at tolerant stage 2-years post-BMT. The dMono was used as a control in comparison. The comparison was based on their RNAseq gene differential expression data sets generated by CLC Genomic Workbench software and the database of well-established signaling pathways from IPA software.
[2]**z-score**: IPA software calculates an 'activation z-score' for each pathway, indicating whether the pathway is likely to be activated or inhibited based on the direction of expression changes in the genes involved, as compared with the IPA database. Pathways were selected if their z-score ≥2 or ≤-2.
[3]**-log p-value**: It's a transformed version of p-value that interprets the significance of pathway. IPA uses a default threshold of -log p-value ≥1.3. The higher value holds greater significance.
[4]**Ratio**: proportion of presented dataset genes in the total number of genes in that pathway within the IPA reference set.
